# Atypical structural snapshots of human cytomegalovirus GPCR interactions with host G proteins

**DOI:** 10.1126/sciadv.abl5442

**Published:** 2022-01-21

**Authors:** Naotaka Tsutsumi, Shoji Maeda, Qianhui Qu, Martin Vögele, Kevin M. Jude, Carl-Mikael Suomivuori, Ouliana Panova, Deepa Waghray, Hideaki E. Kato, Andrew Velasco, Ron O. Dror, Georgios Skiniotis, Brian K. Kobilka, K. Christopher Garcia

**Affiliations:** 1 Department of Molecular and Cellular Physiology, Stanford University School of Medicine, Stanford, CA, USA.; 2 Department of Structural Biology, Stanford University School of Medicine, Stanford, CA, USA.; 3 Howard Hughes Medical Institute, Stanford University School of Medicine, Stanford, CA, USA.; 4 Department of Computer Science, Stanford University, Stanford, CA, USA.; 5 Institute for Computational and Mathematical Engineering, Stanford University, Stanford, CA, USA.

## Abstract

Human cytomegalovirus (HCMV) encodes G protein–coupled receptors (GPCRs) *US28* and *US27*, which facilitate viral pathogenesis through engagement of host G proteins. Here we report cryo–electron microscopy structures of US28 and US27 forming nonproductive and productive complexes with Gi and Gq, respectively, exhibiting unusual features with functional implications. The “orphan” GPCR US27 lacks a ligand-binding pocket and has captured a guanosine diphosphate–bound inactive Gi through a tenuous interaction. The docking modes of CX3CL1-US28 and US27 to Gi favor localization to endosome-like curved membranes, where US28 and US27 can function as nonproductive Gi sinks to attenuate host chemokine-dependent Gi signaling. The CX3CL1-US28-Gq/11 complex likely represents a trapped intermediate during productive signaling, providing a view of a transition state in GPCR–G protein coupling for signaling. Our collective results shed new insight into unique G protein–mediated HCMV GPCR structural mechanisms, compared to mammalian GPCR counterparts, for subversion of host immunity.

## Teaser

The HCMV GPCR-human G protein structures reveal unusual host-pathogen interactions likely critical for HCMV’s pathogenesis.

## INTRODUCTION

Chemokines and chemokine G protein–coupled receptors (GPCRs) orchestrate activation and trafficking of immune cells to fight against invading pathogens (*1*). Herpesviruses have infected and coexisted with human hosts for millions of years, acquiring and co-opting chemokine GPCRs to evade host immunity (*2*). Human herpesvirus (HHV) 5, commonly known as human cytomegalovirus (HCMV), is widespread, with regional infection rates ranging from 45 up to 100% of adults in some developing countries (*3*). HCMV encodes 4 of the 10 HHV-encoded viral GPCRs (vGPCRs), including *US28* and *US27* (*2*, *4*).

From sequence analysis, it is clear that HCMV hijacked human CX3C chemokine receptor 1 (CX3CR1) and evolved gene products US28 and US27, which are roughly as homologous to each other (~30% identity) as they are to CX3CR1. Although these three GPCRs are assumed to have a common ancestor (*4*), host-pathogen coevolution has driven them to completely different chemokine and G protein selectivities (*5*). While CX3CR1 only recognizes CX3C chemokine ligand 1 (CX3CL1, fractalkine) (*6*), US28 promiscuously interacts with both CX3C (hCX3CL1) and CC structural classes of chemokine ligands [e.g., hCCL3, hCCL5, and vCCL2 (vMIP2)] that modulate its intracellular G protein and β-arrestin signaling (*5*, *7*). US28 has also evolved high constitutive activity that is thought to facilitate its function as a chemokine sink to evade host immunity (*8*, *9*). Conversely, US27 is an orphan vGPCR that does not bind to any known chemokine (*5*). Intracellularly, CX3CR1 activates the pertussis toxin-sensitive inhibitory G protein (Gi) upon ligand stimulation (*6*, *10*), similar to the vast majority of chemokine receptors (*11*). On the other hand, US28 has promiscuous intracellular G protein coupling. US28 primarily signals not only through Gq/11 but also via G12/13 and Gi in both ligand-dependent and ligand-independent fashions in certain contexts (*5*, *7*). The G protein specificity of US27 is not yet determined (*5*). Thus, both US27 and US28 represent interesting divergences from human GPCRs, yet their mechanisms for coupling to G proteins remain unclear.

We previously reported the crystal structures of US28 with either wild-type CX3CL1 or a G protein–biased, engineered version of CX3CL1 (CX3CL1.35) (*12*, *13*). The structures, along with a combinatorial chemistry approach to chemokine engineering (*13*), suggested that US28 may have evolved broad ligand promiscuity to enable its “chemokine sink” function (*9*). However, the mechanisms of intracellular signaling via US28 or US27 are not well understood due to the lack of G protein complex structures.

Here, we report a comprehensive cryo–electron microscopy (cryo-EM) structural characterization of US27 and US28 complexes with Gi and Gq/11. In each case, the structural findings are atypical compared to human GPCR–G protein complexes and suggest functional implications necessitated for HCMV pathogenesis and immune evasion. US28 and US27 stably bind to Gi, but they are extremely inefficient as guanine nucleotide exchange factors (GEFs). The structure of US27 shows an occluded ligand binding site that impedes chemokine binding, indicating that US27 exerts its function in a ligand-independent manner. US27 was also coupled with a guanosine diphosphate (GDP)–bound Gi in the inactive conformation, suggesting that US27, as a “Gi decoy,” interferes with host chemokine-Gi signaling. The overall architectures of CX3CL1-US28-Gi and US27-Gi each show two distinct G protein binding modes consistent with curvatures of endosomal membranes, leading us to hypothesize a “G protein sink” function of US28 to sequester G protein from the cell surface via internalization. In addition to the mostly “silent” HCMV GPCR-Gi complexes, we present the structure of signaling-competent CX3CL1-US28-G11 in which the G protein adopts a GDP-bound form. On the basis of molecular dynamics (MD) simulations, we suggest that the GDP-bound CX3CL1-US28-G11 is an intermediate state on the GPCR-mediated G protein cycle, providing a unique example to understand a transition state mechanism of GPCR–G protein coupling for signaling. Collectively, these vGPCR–G protein complexes offer unique views and insights into G protein coupling compared to their mammalian counterparts.

## RESULTS

### US28 and US27 stably interact with Gi but do not cause catalytic activation

**Fig. 1.**
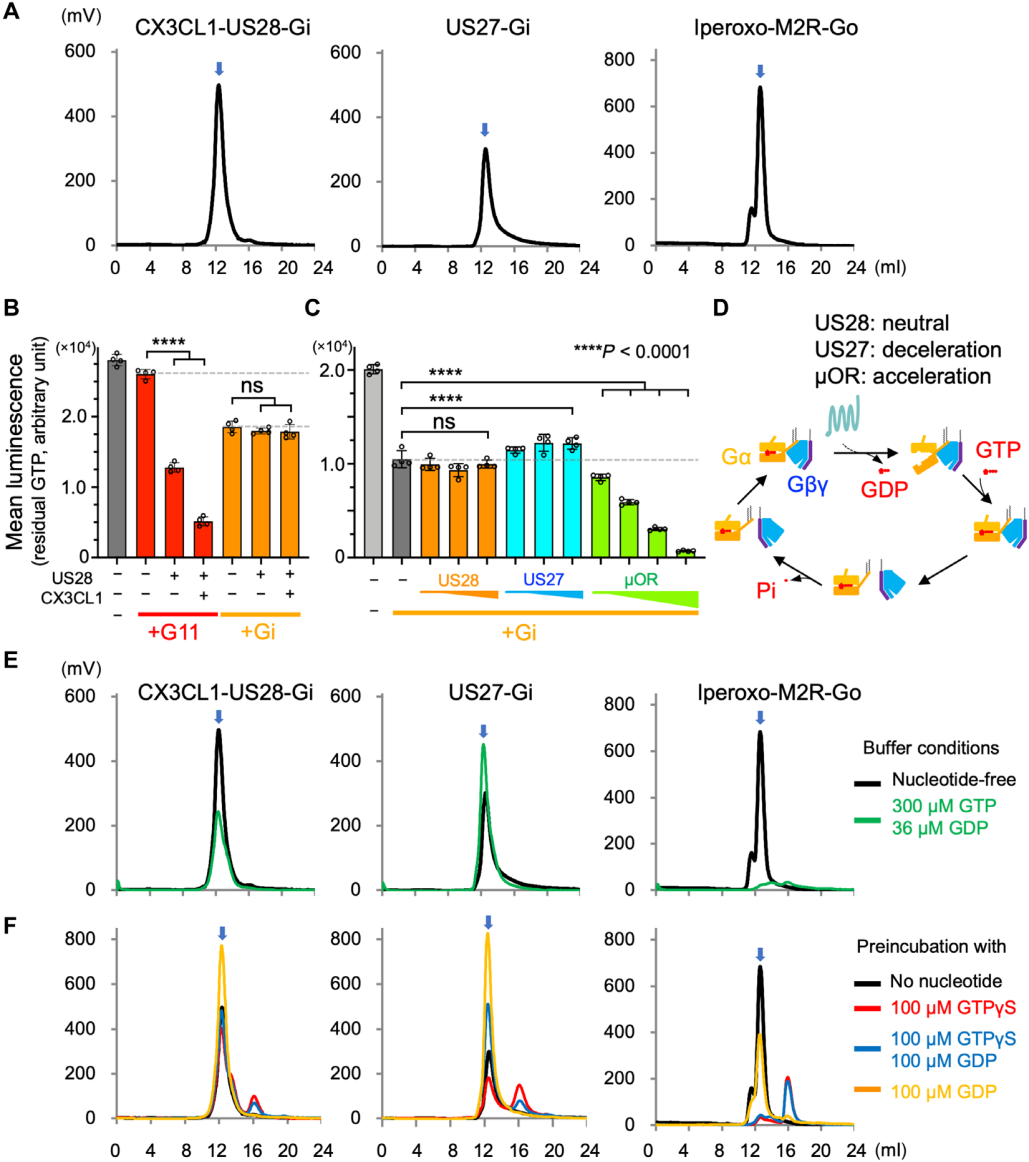
Nonproductive Gi complex formation by US28 and US27. (**A**) FSEC profiles of CX3CL1-US28-Gi, US27-Gi, and iperoxo-M2R-Go without exposure to nucleotides. The elution peaks corresponding to the retention volumes of the complexes are indicated by blue arrows. (**B**) GTP turnover activities of G11 and Gi in the presence or absence of US28 and CX3CL1. The activities were measured for 0.2 μM US28 with or without 0.25 μM CX3CL1 against 0.5 μM G11 or Gi. (**C**) GTP turnover activities of Gi in the presence or absence of US28, US27, and μOR. Activities of μOR were measured at 0.2, 0.5, 1, and 2 μM. Activities of US28 and US27 were measured at 0.2, 0.5, and 1 μM. Gi concentration was 0.5 μM. Gray dashed lines indicate basal activities of G proteins alone. Bars and error bars indicate the mean and SD, respectively, of luminescence values corresponding to the residual amounts of GTP after the guanosine triphosphatase reactions. Data were analyzed by one-way analysis of variance with Tukey’s test, and the statistical significance is shown as *P* < 0.0001 (****). ns, not significant. (**D**) Schematic of G protein cycle. In general, a GEF increases GDP release and GTP binding on G protein, thereby accelerating the G protein cycle. US28 did not affect the basal Gi activities, while US27 decreased the activities of Gi. (**E**) FSEC runs of CX3CL1-US28-Gi, US27-Gi, and iperoxo-M2R-Go with a buffer containing 300 μM GTP and 36 μM GDP (green lines). (**F**) FSEC runs of CX3CL1-US28-Gi, US27-Gi, and iperoxo-M2R-Go after preincubation with either 100 μM GTPγS (red lines), 100 μM GTPγS and 100 μM GDP (blue lines), or 100 μM GDP (orange lines), with a SEC buffer without nucleotides. The elution profiles are shown superimposed with the no-nucleotide controls of (A) (black lines).

To understand the biochemical basis of US28 and US27 coupling with intracellular G proteins, we first examined the interactions between either US28 or US27, prepared recombinantly from human embryonic kidney (HEK) 293S GnTI^−^ cell membranes, and human Gi, which is the most common G protein subtype for immune chemokine GPCRs (*11*). CX3CL1-US28 and US27 (no chemokine added) were incubated with Gi, and then apyrase was added to remove GDP from the solution. Gi binding was confirmed by a receptor pull-down assay (fig. S1A). Size-exclusion chromatography (SEC)–purified complexes were analyzed using tryptophan fluorescence-detection SEC (FSEC) at room temperature (Fig. 1A), showing the stability of the complexes in the nucleotide-free condition. We also characterized signaling directly with a guanosine triphosphate (GTP) turnover assay using purified recombinant proteins (Fig. 1, B to D). Whereas US28 increased basal G11-mediated GTP turnover with further activation by the chemokine agonist CX3CL1, Gi signaling was statistically insignificant from basal Gi activity. Likewise, US27 did not activate Gi but attenuated basal GTP turnover (Fig. 1C). This silent HCMV GPCR-Gi coupling is in contrast to the signaling by the prototypal Gi-coupled GPCR, Met-enkephalin–activated μ-opioid receptor (μOR; Fig. 1C), and suggests that they might form nonproductive vGPCR-Gi complexes.

To investigate this possibility, we first assessed the reliance of the complexes on guanine nucleotide by analyzing the elution profile of CX3CL1-US28-Gi and US27-Gi on FSEC at different concentrations of GDP and GTP [or hydrolysis-resistant guanosine 5′-*O*-(3′-thiotriphosphate) (GTPγS)]. At physiological GTP/GDP concentrations (300 μM GTP and 36 μM GDP) (*14*), US28 and US27 each showed a single peak confirming their superior stability compared to the iperoxo-muscarinic acetylcholine receptor M2 (M2R)–Go complex, which completely dissociated (Fig. 1E). CX3CL1-US28-Gi and US27-Gi were also completely or largely resistant to preincubation with GDP or GTPγS, respectively, whereas M2R-Go showed more substantial dissociation (Fig. 1F).

To measure nucleotide binding kinetics of free Gi and CX3CL1-US28-Gi, we monitored the fluorescence intensity of BODIPY-labeled GTPγS or GDP upon binding to G protein (fig. S1B). CX3CL1-US28-Gi complex formation marginally increased both GTPγS and GDP binding rates compared to free Gi, resulting in the same net GTP/GDP binding preference. This is in stark contrast to the canonical signaling Gi/o complexes such as an agonist-activated M2R-Go that induce faster GTPγS binding but slower GDP binding (*15*), promoting efficient guanine nucleotide exchange. On the basis of these unusual properties of US28-Gi and US27-Gi complexes, we hypothesize that they can form off-pathway, nonproductive GPCR-Gi complexes that function as “Gi decoys” or “Gi sinks” to blunt endogenous Gi signaling by host chemokine GPCRs, thereby impairing the sensitivity of HCMV-infected host cells to chemokines.

### Cryo-EM structures of the CX3CL1-US28-Gi and US27-Gi complexes

**Fig. 2.**
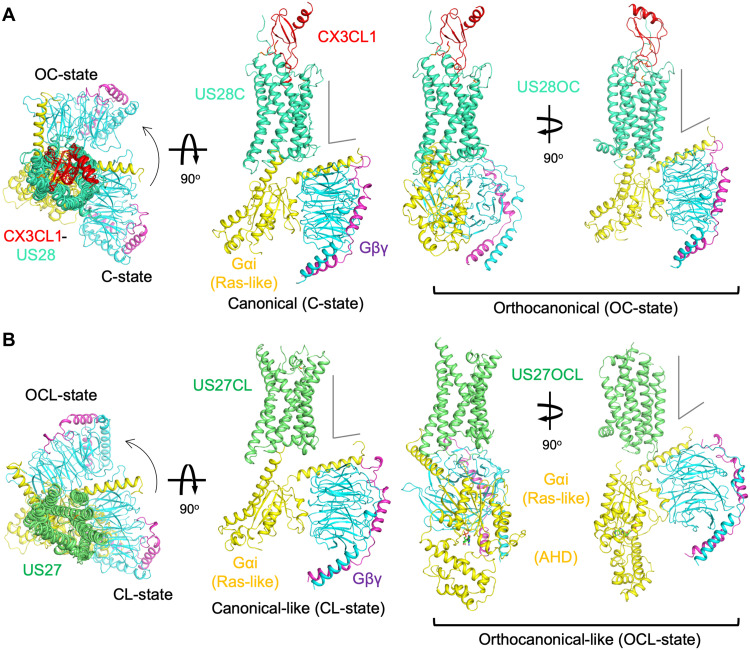
The structures of CX3CL1-US28-Gi and US27-Gi complexes. Structures of (**A**) CX3CL1-US28-Gi and (**B**) US27-Gi. The left panel shows the top-down view of each of the US28 and US27 complexes in two distinct states, the C- and OC-states for US28 (US28C and US28OC) and the CL- and OCL-states for US27 (US27CL and US27OCL), aligned using the receptor region to show the Gi rotations indicated by the curved arrows. The right panels show the side views of the four structures to compare their vGPCR:Gi binding modes and the Gi tilt toward the membrane plane. The structures are displayed as cartoon representations with disulfide-forming cysteines shown as sticks. Each chain is colored in red (CX3CL1), green-cyan (US28), lime (US27), yellow (Gαi1), cyan (Gβ1), and purple (Gγ2). ScFv16 structure is omitted for clarity.

With the aid of the G protein–stabilizing antibody scFv16 (*15*), we performed cryo-EM analysis of the CX3CL1-US28-Gi and US27-Gi complexes. The CX3CL1-US28-Gi complex showed two particle classes with distinct US28:Gi binding modes (Fig. 2A, figs. S2 and S3, and table S1). The three-dimensional (3D) reconstruction of one class is similar to many other GPCR–G protein complex structures reported so far. Because of the similarity to a large majority of GPCR–G protein complexes, we term this conformation the canonical-state (C-state). The other class yielded a complex in which Gi is rotated approximately 90° around the transmembrane (TM) axis relative to the C-state, which we term the orthocanonical-state (OC-state). For class A GPCRs, a “noncanonical” GPCR–G protein docking mode has been thus far only reported for neurotensin receptor 1 (NTSR1)–Gi complex (*16*), where the Gi protein is rotated approximately 45° around the TM axis relative to the C-state.

We also carried out cryo-EM analysis for US27-Gi (Fig. 2B, figs. S2 and S4, and table S1), which showed an inhibition of Gi activity in vitro (Fig. 1B). Similar to the US28 complexes, we captured two states with analogous G protein rotation: the canonical-like state (CL-state) and orthocanonical-like state (OCL-state). The CL-state of US27-Gi resembles the C-state of CX3CL1-US28-Gi at first glance; however, the cryo-EM map indicated that the α helix 5 (α5) of Gαi was not fully inserted into the cytoplasmic core of US27 compared to the C-state CX3CL1-US28-Gi and other canonical GPCR–G protein complex structures. Because of the unstable interaction modes of US27-Gi, the cryo-EM map of the TM region, especially the TM4 side with no direct contact to Gαi, was not of as high quality as the CX3CL1-US28-Gi maps that have full α5 insertion into the receptor’s cytoplasmic core, stabilizing the receptor conformation and orientation relative to Gi (figs. S3 and S4).

In the OCL-state, the position of Gi rotates approximately 90° with respect to the CL-state of US27-Gi, analogous to the OC-state CX3CL1-US28-Gi. Unexpectedly, we observed the closed α-helical domain (AHD) in the OCL-state with GDP bound between the Ras-like domain and AHD (figs. S2 and S4), suggesting that Gi is in its inactive state. We carried out apyrase treatment for all the complexes in this study to align the sample preparation condition with prior structural studies of GPCR–G protein complexes. This would bias the complex population to nucleotide-free states. Nonetheless, we still obtained the GDP-bound OCL-state. To improve the OCL-state map, we prepared the complex with no apyrase treatment and collected datasets in the presence or absence of 100 μM GDP (see Materials and Methods and fig. S2C). In the presence of a threefold molar excess of GDP (100 μM), all the intact complex particles were in the OCL-state with stable AHD density. However, in both the conditions meant to increase the population of the OCL-state, we observed more dissociation of the complex, indicating an inherent instability of the OCL-state to adverse effects during cryo-EM grid preparation, as well as limiting the reconstructed map at modest resolution. Analysis of the map revealed the binding interface to be even less extensive than in the CL-state US27 complex, with only ambiguous cryo-EM density connecting Gαi-α5 and US27-7TM (fig. S4G), but the resulting map enabled docking of the US27 model from the CL-state complex and the GDP-bound Gi heterotrimer [Protein Data Bank (PDB) ID: 1GP2]. The unusual OCL-state complex suggests that US27 trapped Gi in the inactive state independent of the GPCR-mediated guanine nucleotide release and that the OCL-state of US27-Gi and likely the analogous OC-state CX3CL1-US28-Gi are nonproductive complexes that may serve as decoy forms to slow GDP release. Notably, in both the OC- and OCL-states of the vGPCR-Gi complexes, Gi heterotrimers are highly tilted toward the membrane plane (Fig. 2).

### The structure and Gi binding mode of the orphan vGPCR US27

**Fig. 3.**
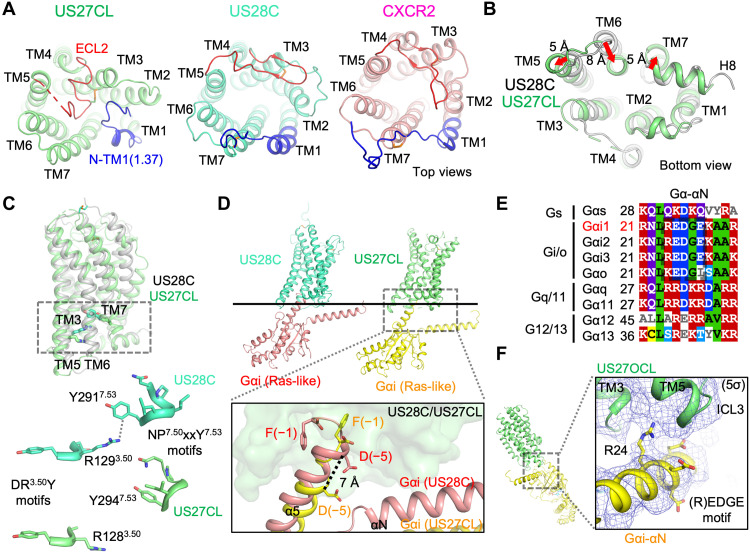
Structural analysis of the nonproductive US27-Gi complex. (**A** to **C**) Structural comparison among HCMV GPCRs and an endogenous chemokine receptor in Gi-bound states. (A) Cartoon representation of Gi-bound US28 (C-state, green-cyan), US27 (CL-state, lime), and human CXCR2 (PDB ID: 6LFO, light pink) from the top view. The N terminus to TM1 residue 1.37 (generic number) region and the ECL2 region are colored in blue and red, respectively, to highlight US27’s extracellular capping structures. (B) Superimposition of the C-state US28 (gray) and the CL-state US27 (lime) from the bottom view, showing the difference of the relative positions of TM6 in the 7TM bundle. (C) Different configuration of the signature motifs in GPCR activation between C-state US28 (gray cartoon in superimposition, and green-cyan) and CL-state US27 (lime). The distances between R^3.50^ and Y^7.53^ are ~4 and ~12 Å in US28 and US27, respectively. (**D** to **F**) Structure of US27-Gi in the CL- and OCL-states. (D) Side-by-side comparison and superimposition of US28-Gαi in the (C-state, US28: green-cyan, Gαi: light pink) and US27-Gαi (CL-state, US27: lime, Gαi: yellow) structures to show the shallow Gαi-α5 engagement into US27. (E) Multiple sequence alignment of N-terminal regions of Gα proteins, which corresponds to another major US27:Gαi-αN interface in the OCL-state. Sequence alignment was done on the Clustal Omega server (ebi.ac.uk/Tools/msa/clustalo/), and the result was displayed by MView (ebi.ac.uk/Tools/msa/mview). (F) An interface between US27 and Gαi-αN in the OCL-state.

In contrast to US28 (*12*, *13*), US27 exhibits an inactive-like conformation despite a stable complex formation with Gi protein. The global Cα root mean square deviation (RMSD) between the CL-state US27 and the C-state US28 is ~3.0 Å with notable differences in the extracellular region. First, ECL2 of US27 folds back into the orthosteric ligand-binding site to occupy the pocket. Second, TM1 of US27 has a sharp turn at the extracellular end to cover ECL2, thereby stabilizing the insertion of loops into the orthosteric ligand-binding site. These structures together act as a plug to fill the extracellular “chemokine-binding pocket” defined for viral and human chemokine receptors (Fig. 3A and fig. S4). The ECL2 plug and bending TM1 lid help to explain how US27 has evolved from the ancestral chemokine GPCR to prevent binding to any chemokine ligand. Another prominent feature is an inward position of US27-TM6, which is a signature of the inactive-state GPCRs (*17*), where the intracellular end is ~8 Å inward compared with the C-state CX3CL1-US28-Gi complex (Fig. 3B). The inwardly placed TM6 forces TM5 and TM7 outward, and the closed intracellular pocket yielded ~12 Å distance between R128^3.50^ and Y294^7.53^ [superscripts denote generic numbering for class A GPCRs (*18*)] (Fig. 3C), the residue pair known to undergo conformational rearrangement to form an active state GPCR. While maintaining the ability to bind Gi (Fig. 1A and fig. S1A), these features imply US27 evolved to be “constitutively inactive” for G protein signaling independent of ligand binding.

US27 binds Gi through highly tenuous molecular interfaces both in the CL- and OCL-states (figs. S4 and S5). In the CL-state, US27 exclusively binds Gαi-α5 with a substantially smaller molecular interface of ~650 Å^2^ (fig. S5), compared to canonical GPCR–G protein complexes having ~1000 Å^2^ GPCR:Gα interfaces via multiple interaction sites. This unique US27:Gi binding mode results in an approximately 1.3 helical turn (~7 Å) downward transition of Gαi-α5 in comparison with the C-state CX3CL1-US28-Gi (Fig. 3D). F(−1) to C(−4) of Gαi forms a loop structure similar to other GPCR-Gi complexes including CX3CL1-US28-Gi, but the loop is more extended and only the F(−1) side chain is inserted into the intracellular core of US27 and primarily contacts the TM6 side, with the remaining Gαi-α5 positioned near the concave surface formed by TM3, TM5, and TM6 of US27 (Fig. 3D). This suboptimal binding mode supports poor guanine nucleotide exchange activities seen by US27 (Fig. 1C).

In the US27 OCL-state, the G protein rotation and tilt yield completely distinct molecular interfaces from the CL-state. Low-resolution receptor docking indicates that US27-ICL3 interacts with Gαi-αN and that US27-ICL2, flexible in the CL-state, makes a new contact with Gβ. We note that the C-terminal region of Gαi is not modeled as there is a blurred cryo-EM density observed between F(−1) and D(−5) at the inner surface of TM6, indicating a partial unfolding of α5 helix (fig. S4G). The resolved US27:Gαi interface is formed by TM3, TM5, and ICL3 of US27, with the density approaching the Gαi specific Arg^24^-Glu^25^ of the (R)EDGE motif of αN (Fig. 3, E and F) and possibly contributing the binding selectivity of US27 toward Gi over Gs or Gq (fig. S6).

### Structure of CX3CL1-US28-Gi in its C- and OC-states

**Fig. 4.**
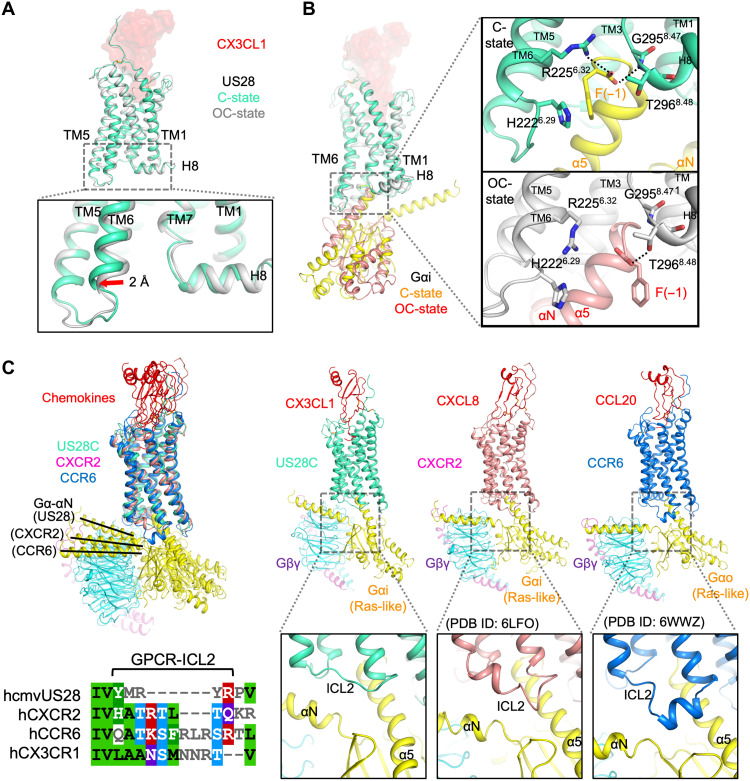
Structure of US28-Gi in the C- and OC-states. (**A**) Superimposition of CX3CL1-US28 structures in the C-state (green-cyan US28) and the OC-state (gray US28), showing different TM6 conformations. (**B**) The US28:Gαi interfaces at the α5 ends in the two states. CX3CL1 is shown as transparent surface representations (red), and US28 and Gαi (yellow, +US28C; light pink, +US28OC) are shown as cartoon representations, with the focused residues displayed as sticks. (**C**) Structural comparison among the C-state CX3CL1-US28-Gi, human CXCL8-CXCR2-Gi (PDB ID: 6LFO), and human CCL20-CCR6-Go (PDB ID: 6WWZ), aligned with the receptor regions colored in green-cyan (US28), light pink (CXCR2), and blue (CCR6). The left panel shows the superimposition, and the right three panels are the side-by-side comparison of the three chemokine-chemokine receptor-Gi/o complex structures shown as cartoon models. The chemokines Gα, Gβ, and Gγ are colored red, yellow, cyan, and purple, respectively. The ICL2 sequence alignment, made on the PROMALS3D server (prodata.swmed.edu/promals3d) using the three structures (US28, CXCR2, and CCR6) and all four sequences of GPCRs as inputs, is also shown with MView.

Despite the large difference in the relative orientations of US28 and Gi, the overall structures of both the ligand-receptor complex and of the Gi heterotrimer are similar between the C- and OC-states. The US28-7TM structures adopt the active-like conformation in both cases with a Cα RMSD of ~0.6 Å. Both the C- and OC-states have extensive interactions with interface areas to Gαi of ~1150 and ~1110 Å^2^, respectively, and additional contacts to Gβ with ~260 and ~180 Å^2^ interfaces, respectively (fig. S5). The Gβ interactions are comparable to the interface area seen in the noncanonical state of the NTSR1-Gi complex (*16*). The common Gαi-Gβγ bridging by US28 might negatively affect the GEF efficiencies observed, both at the C- and OC-states. A small but noticeable difference is observed in the TM6 position. The intracellular end of TM6 in the US28 C-state is displaced ~2 Å more outward than the OC-state US28 and US28 bound to G protein mimetic Nb7 (Fig. 4A and fig. S7, A and B) (*12*, *13*). The TM6 angles, along with the overall US28-Gi docking modes, indicate that the C-state resembles more closely the genuinely active state, and the OC-state likely represents an “off-pathway” decoy conformation. Given CX3CL1 serves as a partial agonist while CCL5 works as a full agonist for G protein signaling (*13*), CCL5-bound US28 may alter the preference in favor of the genuine “active state” that would be slowly transitioned from the C-state.

While the individual structural components are folded into similar conformations, the interaction modes differ markedly between the two states due to the G protein rotation (Fig. 4B and fig. S7C). For instance, the F(−1) side chain and the C-terminal carboxyl group of Gαi are bridging TM6 and the TM7-H8 turn of US28 in the C-state, similar to a majority of endogenous C-state GPCR-Gi/o complexes, while only the carboxyl group is mediating the interaction in the OC-state (Fig. 4B). The C-state complex, but not the OC-state complex, also maintains many conserved interactions between the receptor’s intracellular loops and Gi protein (fig. S7C). US28-ICL3 is inserted between α5 and the α4-β6 loop of Gαi in the C-state, while it interacts with α5 and the β2-β3 hairpin of Gαi in the OC-state, where the other end of the β2-β3 sheet is another important regulator of GDP release and GTP binding during the G protein cycle. Apart from the core α5 interactions, US28-ICL2 runs in parallel with Gαi-αN in the C-state, whereas the same loop makes extensive contact with Gβ in the OC-state. US28-ICL1 plays a major role in forming the Gβ interface in the C-state, while it interacts only with α5 in the OC-state. The residue-residue interactions are diagrammed in fig. S7D.

Superimposition of the C-state CX3CL1-US28-Gi and reported human chemokine receptor structures, CXCL8-CXCR2-Gi (*19*) and CCL20-CCR6-Go (*20*), shows that the C-state US28 aligns well with the active CXCR2 and CCR6 with the overall RMSDs of ~1.2 and ~1.5 Å, respectively (Fig. 4C). However, the US28-ICL2 is substantially diverged from the Gi-specific human homolog CX3CR1 (Fig. 4C and fig. S8) and is shorter than endogenous chemokine receptors in general. In the case of human chemokine receptors, the longer ICL2 forms a partial α helix to contact the Gα subunit more deeply, resulting in a small rotational difference in the G protein binding mode. This ICL2 deviation might contribute to the unique G protein coupling promiscuity of US28.

### Shared and unique structural features of Gαi protein coupled to HCMV GPCRs

**Fig. 5.**
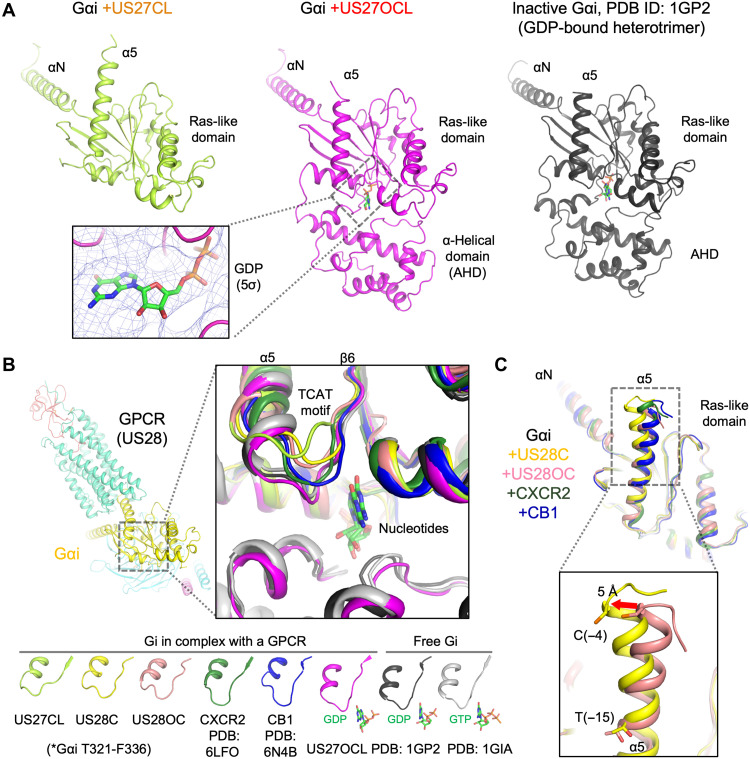
Structure of Gi protein in complex with HCMV GPCRs. (**A**) Structural comparison between Gαi bound to US27 (limon: CL-state, pink: OCL-state) and in the inactive Gi heterotrimer (dark gray, PDB ID: 1GP2). A close-up view of the GDP observed between the Ras-like domain and AHD of Gαi is in the outlined box with the cryo-EM density at 5σ contour level. (**B**) Structural comparison of the TCAT motifs of Gαi. Cartoon models are colored in limon (+US27CL), yellow (+US28C), light pink (+US28OC), forest (+CXCR2, PDB ID: 6LFO), blue (+CB1, PDB ID: 6N4B), pink (+US27OCL), dark gray (GDP-bound heterotrimer, PDB ID: 1GP2), and gray (GTP-bound, PDB ID: 1GIA). Nucleotides are shown as stick models. (**C**) Superimposition of Gαi bound to US28C (yellow), US28OC (light pink), CXCR2 (forest), and CB1 (blue).

The G protein structures in the complexes are consistent with the inefficient GEF activities by the HCMV GPCRs. The most notable feature is that the OCL-state US27 holds the GDP-bound inactive Gi (Fig. 5A) with less-extended Gαi-α5. Except for this difference, the Gαi structure is basically similar between vGPCR-Gi and endogenous mammalian GPCR-Gi complexes. However, there is a notable difference in the β6-α5 loop containing the TCAT motif (T324, C325, A326, and T327 in Gαi1) that forms the nucleotide-binding pocket (Fig. 5B). Canonical GPCR–G protein binding induces extension of Gα-α5 associated with structural rearrangement of the β6-α5 loop, thereby altering interactions with the guanine ring of GDP and GTP. Compared to other canonical GPCR-Gi complexes, such as CXCR2-Gi and CB1-Gi, we observed that the β6-α5 loop is pulled away from the nucleotide-binding pocket toward the receptor side in the nucleotide-free state HCMV GPCR-Gi complexes. This unique conformation of the β6-α5 loop may partly explain the atypical guanine nucleotide binding properties of CX3CL1-US28-Gi and US27-Gi. We also observed a difference in the Gαi-α5 conformation in the CX3CL1-US28-Gi complexes (Fig. 5C). In the C-state US28 complex, the Gαi-α5 bends at around T(−15), whereas most other GPCR–G protein complexes have a straight α5 conformation. This yielded a ~5-Å shift at C(−4) toward the αN side, stabilized by US28 contacts to Gβγ, compared to the OC-state US28 complex and other endogenous GPCR–G protein complexes. Notably, a release of this bending tension would make the straight α5 conformation, moving US28 away from Gβγ to yield the binding mode expected for the genuinely active state.

### MD simulations of CX3CL1-US28-Gi support the Gi sink hypothesis

**Fig. 6.**
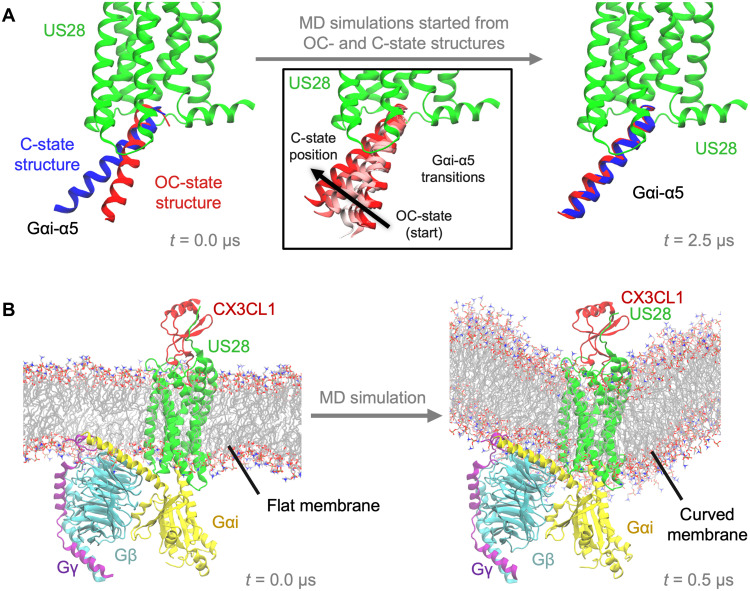
MD simulations of the CX3CL1-US28-Gi complex. (**A**) Unbiased simulations of the complex started from both the C-state and OC-state structures converge to the C-state. Positions of the Gαi-α5 helix in the two structures are shown at the left (aligned on US28, shown in green). Positions adopted by the same helix in representative simulations started from the C-state (blue) and OC-state (red) structures are shown at the right. The remainder of Gi is included in the simulations but not shown here, for simplicity (see fig. S9 for a view of the entire complex). The middle panel shows the smooth transition of Gαi-α5 from the OC-state to the C-state in a representative simulation; simulation frames are shown every 250 ns and color-coded from red to white to red by simulation time from 0.5 to 2.0 μs. (**B**) In simulations where the complex is restrained to its OC-state structure, the lipid membrane adopts a curved shape. Lipids are shown with lines corresponding to bonds between atoms (C, gray; O, red; N, blue; P, tan).

To better understand the two experimentally observed Gi binding modes with different G protein tilts (Fig. 2), we carried out unbiased, all-atom MD simulations of CX3CL1-US28-Gi in a lipid membrane, using both the C-state structure and the OC-state structure as starting points (Fig. 6 and fig. S9). We found that simulations started from the OC-state structure typically transitioned to the C-state, while those started from the C-state structure remained in the C-state. In particular, Gαi-α5 generally adopted the same C-state position in simulations started from either structure (Fig. 6A and fig. S9, A and B). Gαi-α5 typically transitioned smoothly and spontaneously from its OC-state position to its C-state position. Despite substantial conformational flexibility, the rest of Gi tended to rotate in the same direction and tilt away from the membrane plane, adopting conformations resembling the C-state structure (fig. S9, C and D).

Our initial simulations, which were performed in a flat lipid membrane, suggest that the C-state is favored in flat membranes. The tilt of Gi toward the membrane plane in the OC-state structure (Fig. 6) suggests that a convex membrane would be a more favorable environment for the OC-state. To probe this hypothesis, we performed additional simulations in which we restrained the complex to the OC-state structure or the C-state structure. When the complex was restrained to its OC-state structure, the lipid membrane adapted by changing to a shape puckered out toward the extracellular side (Fig. 6B). In contrast, the membrane remained flat when the complex was restrained to its C-state structure. Moreover, the continuum of Gαi-α5 positions along the transition pathway between the two states (Fig. 6A) allows the CX3CL1-US28-Gi complex to flexibly adapt to a wide range of intermediate membrane curvatures such as these of endosomal vesicles (*21*, *22*). The US27-Gi complex should be compatible with a similarly broad range of membrane curvatures, given its flexible nature and the even longer distance between the receptor and the Gi core.

Thus, although the high tilt observed in the OC-state US28 complex and the OCL-state US27 complex are exaggerated compared to what might be observed in endosomal vesicles due to the round and smaller surface of the detergent micelle, the MD simulations hint at how US28 and US27 could bind Gi while embedded in endosomal membranes, further supporting a “Gi sink” hypothesis. US28 and US27 are constitutively and preferentially localized in endosomes, and the proportion of the endosomal US28 is enhanced upon the addition of CX3CL1 (*23*, *24*). Although US28 is constitutively coupled with β-arrestin for signaling (*13*, *25*), it has been documented that the receptor can localize in endosomes through β-arrestin–independent internalization pathways (*26*, *27*). Considering their prolonged endosomal localization, the tilted Gi conformations in the OC- and OCL-state structures might enable US28 and US27 to trap Gi protein at the endosome without catalytic activation, segregating it from cell surface GPCRs such as chemokine receptors. This Gi sink function would facilitate depletion of the available Gi protein from the cell surface, thereby silencing chemokine signaling, which is largely mediated by Gi signaling and β-arrestin.

### Mechanism of Gq/11 activation by US28

**Fig. 7.**
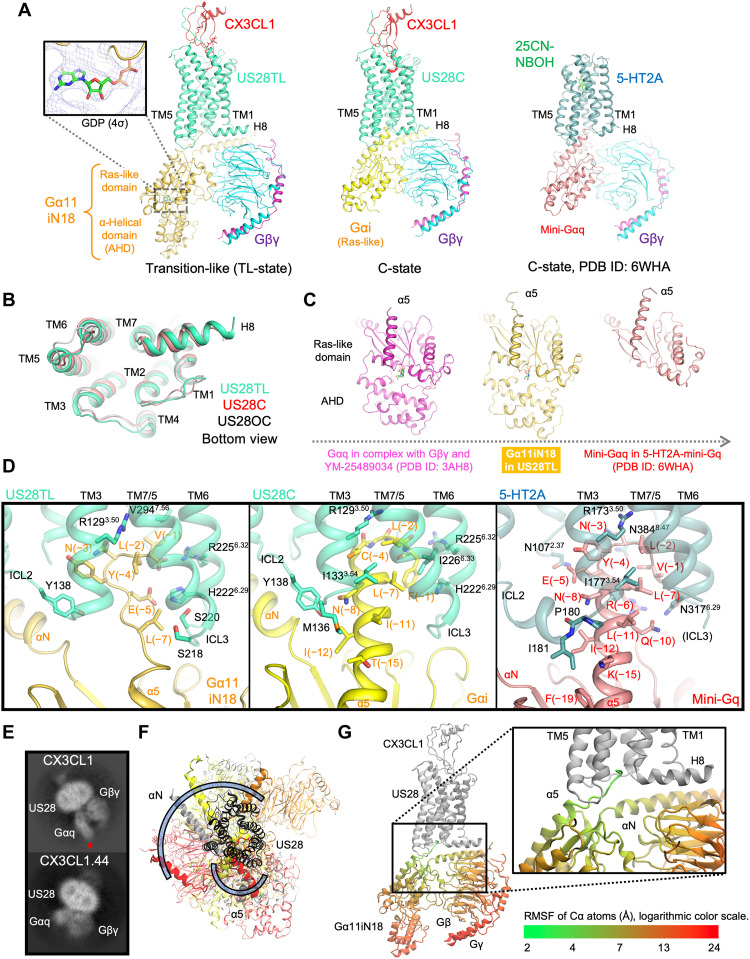
Structural analysis of Gq/11 coupling by US28. (**A**) Cryo-EM structures of the TL-state CX3CL1-US28-G11iN18 (US28TL, left), the C-state CX3CL1-US28-Gi (US28C, middle), and the C-state (25CN-NBOH)–(5-HT2AR)–mini-Gq (right, PDB ID: 6WHA). Each protein chain is colored in red (CX3CL1), green-cyan (US28), turquoise (5-HT2AR), yellow-orange (Gα11iN18), yellow (Gαi), light pink (mini-Gαq), cyan (Gβ1), and purple (Gγ2). The GDP bound to Gα11iN18 is shown in close up with cryo-EM density at the contour level of 4σ. (**B**) Superimposition of the CX3CL1-US28-G protein complexes in the TL-state (green-cyan), C-state (light pink), and OC-state (US28OC, gray). (**C**) Structural comparison among Gα11iN18 in the TL-state US28 complex (yellow-orange, middle), Gαq in the inactive Gq heterotrimer (left, pink, PDB ID: 3AH8), and active mini-Gq bound to (25CN-NBOH)–(5-HT2AR) (right, light pink, PDB ID: 6WHA). The α5 regions are outlined for clarity. (**D**) Close-up views of the G protein–α5 interacting with (left) US28TL, (middle) US28C, and (right) (25CN-NBOH)–(5-HT2AR). (**E**) Representative 2D class averages for (top) CX3CL1-US28-Gq and (bottom) CX3CL1.44-US28-Gq with no scFv16. The Gα-AHD density observed for CX3CL1-US28-Gq is indicated by the red arrow. (**F** and **G**) MD simulations for CX3CL1-US28-G11iN18. (F) MD simulations for CX3CL1-US28-G11iN18 illustrating large-scale motions of Gα11iN18 in the TL-state. Example top views on the G protein from 3 of 12 independent simulations are shown (yellow, orange, and red) overlayed on the starting model (gray), with all structures aligned on the receptor. The α5 and αN regions are highlighted, and US28 is shown in black. Blue curves show the range of motion of α5 and αN. (G) Root mean square fluctuation (RMSF) in MD simulations of the CX3CL1-US28-G11iN18 complex. The average RMSF values over all 12 independent simulations are indicated by the logarithmic heatmap colored on Gα11iN18 from green (2 Å, more stable) via yellow (7 Å, intermediate) to red (25 Å, more flexible).

US28 is a promiscuous GPCR that couples with multiple Gα protein subtypes (*5*, *7*). To investigate this behavior, we sought to solve the active structure of a Gq/11 family protein bound to CX3CL1-US28 as it catalyzed nucleotide turnover of G11 in vitro (Fig. 1B). To prepare the complex, we used a chimeric Gα11 termed Gα11iN18 (G11iN18 as a heterotrimer) where the amino terminal region of Gα11 is replaced with the primary scFv16 binding epitope from the αN residues of Gαi1 (*15*). We obtained a stable complex after apyrase treatment to hydrolyze released GDP (fig. S10), resulting in a 3D reconstruction with sufficient quality to dock the published structures of each component and for structure refinement (fig. S11). Unexpectedly, although CX3CL1-US28 promoted nucleotide exchange of G11 (Fig. 1B), the cryo-EM structure of CX3CL1-US28-G11iN18 revealed that GDP remained bound with the AHD closed (Fig. 7A). This discrepancy between the functional assay and the cryo-EM structure could be due to the biochemical and cryo-EM specimen preparation processes where most of the nucleotide-free complexes might have dissociated such that only GDP-bound complex remained intact. The overall GPCR–G protein docking mode was reminiscent of the nucleotide-free CX3CL1-US28-Gi and (25CN-NBOH)–5-HT_2A_ serotonin receptor (5-HT2AR)–mini-Gq in their C-states (*28*) (Fig. 7A) and other active GPCR–G protein complexes. Similar to the C-state CX3CL1-US28-Gi, TM6 is in the fully open conformation (Fig. 7B). On the basis of these features, we surmised that the obtained GDP-bound CX3CL1-US28-G11iN18 complex may resemble a transition-like state (TL-state) of the complex before the full GPCR–G protein engagement. It is also possible that the cryo-EM structure captured an off-pathway, nonproductive GPCR–G protein complex as seen in the OCL-state US27-Gi complex. However, this scenario is unlikely because US28 triggers nucleotide turnover of Gq/11 (Fig. 1B) (*13*).

In the CX3CL1-US28-G11iN18 complex, the structure of the G protein heterotrimer resembled the crystal structure of the heterotrimeric Gq bound to GDP and an inhibitor YM-25489034 (PDB ID: 3AH8), yielding a Cα RMSD of ~1.8 Å. The Gα structures were also similar to each other with a Cα RMSD of ~1.5 Å. However, the Ras-like domain and AHD were slightly more packed for Gαq than Gα11iN18 presumably due to the binding of YM-25489034 to bridge these domains (Fig. 7C). Another notable feature is the conformation of Gα11iN18-α5, where the terminal residues adopt a loop structure to reach the intracellular pocket of the CX3CL1-activated US28 (Fig. 7, C and D), which also appears to be intermediate between the inactive and active states. This unique α5 interaction mode has not been observed in other GPCR–G protein complex structures (Fig. 7D). Notably, in the TL-state complex, US28-ICL2 does not interact with the partially formed α helix region of Gα11iN18-α5, in contrast to the C-state US28-Gi complex and the (25CN-NBOH)–(5-HT2AR)–mini-Gq complex. A recent mass spectroscopic analysis showed that the receptor ICL2 region plays a pivotal role in the GDP release during the full α5 engagement for β2-adrenergic receptor-Gs (*29*). The lack of ICL2 engagement in the TL-state further supports the interpretation that the current CX3CL1-US28-G11iN18 complex represents an initial G protein recognition state.

To experimentally confirm that the obtained TL-state structure is not an artifact from the chimeric G protein or the G protein–stabilizing scFv16, we obtained cryo-EM 2D class averages and a low-resolution 3D reconstruction using the complex with wild-type Gq protein (Fig. 7E and fig. S10C). Note that we used Gq, not G11, as the Gq complex was more stable. We still observed evidence for GDP-bound complexes, consistent with the CX3CL1-US28-G11iN18 structure with scFv16. We also performed analogous experiments and analysis using an engineered version of CX3CL1 termed CX3CL1.44 (*13*). In CX3CL1.44, the N-terminal amino acid sequence (QHHGVTK) of the parental CX3CL1 ligand is replaced by VRPHINN, which yields more potent Gq/11-mediated calcium signaling while showing weaker β-arrestin–mediated cell migration on HEK293 cells (*13*). As expected, the cryo-EM 2D classes showed that the surviving holocomplexes were predominantly in the nucleotide-free state (Fig. 7E and fig. S10C), suggesting that the more efficacious agonist promoted GDP release and stabilized the putative signaling state. These results further support our interpretation that the obtained GDP-bound structure is a natural intermediate state.

Last, MD simulations of the GDP-bound CX3CL1-US28-G11iN18 complex in a lipid bilayer (Fig. 7, F and G, and fig. S12, A and B) show that the G protein is highly mobile with respect to the receptor. Over the course of the simulations, the C terminus of Gα11iN18 remained within the transducer binding pocket, anchoring the G11iN18 heterotrimer to CX3CL1-US28. The rest of the G protein underwent large-scale motions, with the flexible uncoiled region of Gα11-α5 acting as a ball joint for the CX3CL1-US28-G11iN18 complex (Fig. 7, F and G, and fig. S12A). In contrast, in simulations of CX3CL1-US28-Gi in the C-state, the G protein exhibits much less mobility (fig. S12B), and the receptor forms more stable contacts with the G protein. Furthermore, we performed simulations of the complex after removing the bound GDP, and we found that GDP removal destabilized the CX3CL1-US28-G11 TL-state complex (fig. S12, C and D). Across GPCR–G protein complexes, most experimentally determined conformations resemble the C-state. These observations suggest that the final state of the binding process in CX3CL1-US28-G11iN18 likely resembles the C-state while the TL-state is an intermediate state.

## DISCUSSION

Our structural studies have revealed an unexpected diversity in how G proteins can bind to the two HCMV GPCRs, US28 and US27. In each case, the complexes reveal atypical features compared to mammalian GPCRs and, in this way, expand our insights into the spectrum of ways that GPCRs can engage and couple to G proteins. The extent that the unusual structural features are linked to the biological functions of HCMV remains to be determined. But in some cases, particularly the nonproductive US28 and US27 complexes and the ligand-independent actions of US27, the results strongly infer G protein decoy functions to blunt the host immune response orchestrated through induction of host chemokines (*30*). This adds a twist to viral decoy mechanisms to subvert host immunity, along with US28’s chemokine sink function.

Intriguingly, having the same ancestral chemokine receptor as US28, US27 had the occluded extracellular “ligand-binding” pocket as seen in Epstein-Barr virus (EBV)–encoded GPCR BILF1 (*31*). However, in contrast to constitutively active BILF1, US27-7TM is folded into the inactive conformation with a narrow intracellular pocket coupling with Gi. The constitutively inactive and active 7TM conformations of US27 and BILF1, respectively, suggest distinct evolutionary hallmarks of the vGPCR-mediated immune evasion mechanism between HCMV and EBV.

Among the five structures described here, the C-state CX3CL1-US28-Gi complex most resembles canonically active GPCR–G protein complex structures reported so far. The other state, the OC-state CX3CL1-US28-Gi complex, shows a completely distinct GPCR–G protein docking mode from the C-state complex with the 90° G protein rotation and high-tilt toward the membrane plane. MD simulations suggested that the complex can continuously transition between the C- and OC-states upon changes of membrane curvature while maintaining similar interface areas of US28 with Gαi and with Gβ. Adopting intermediate conformations between the two binding modes, the CX3CL1-US28-Gi complex can accommodate the polydisperse membrane curvatures of endosomal vesicles and cell surfaces. Similarly, two distinct engagement modes with more tenuous interactions were observed in the structures of US27 bound to Gi. In the CL-state, nucleotide-free Gi is flexibly anchored to the receptor core only via the tip of Gαi-α5. The OCL-state is more similar to the OC-state of the US28 complex in terms of the GPCR–G protein rotation and tilt but traps the GDP-bound inactive Gi heterotrimer. In the HCMV GPCR-Gi structures, we observed that the two US28 complexes and the OCL-state US27 complex bridge Gα and Gβ of nucleotide-free Gi and GDP-bound Gi, respectively, with perceptible interfaces between the receptors and Gβ. This gentle receptor-mediated stabilization of the Gα-Gβγ complex might slow the GTP binding to the CX3CL1-US28-Gi complex and GDP release from the US27-Gi complex, analogous to the stabilization mediated by scFv16 (*15*), possibly resulting in the atypical biochemical and signaling properties observed.

We also captured the GDP-bound TL-state CX3CL1-US28-Gq/11 complex that appears to be an initial GPCR–G protein engagement state preceding the GDP release. The TL-state complex also has a continuous interface to Gα-Gβγ, indicating that this feature may also be important for the high-affinity initial engagement between the receptor and G protein. Upon transition to the genuinely active state, the receptor should more exclusively interact with Gα for efficient signaling. The nucleotide-free GPCR–G protein complex should be energetically stable for typical GPCR–G protein combinations. Earlier crystal and high-resolution cryo-EM structures are likely biased toward more rigid and stable binding modes, missing out on the more dynamic states. The atypical binding modes described here might be the consequence of the unique evolutional strategy of HCMV, making multiple metastable GPCR–G protein coupling states more readily accessible, allowing us to visualize previously unseen structures.

Similar to EBV, HCMV is a ubiquitous virus that causes lifelong latent, asymptomatic infection in healthy individuals (*32*). However, for immunologically weak patients such as fetuses and newborns, HCMV can cause severe symptoms. HCMV-encoded viral proteins mediate host immune evasion, and US28 plays a major role in the establishment of the viral latency and reactivation (*7*). Hence, a mechanistic understanding of HCMV persistence is important for the treatment and prevention of HCMV infection and HCMV-mediated diseases (*33*). Our study opens up an opportunity to further explore the immune evasion mechanisms used by HCMV.

## MATERIALS AND METHODS

### Expression and purification of CX3CL1-US28, CX3CL1.44-US28, *apo* US28, and US27

The chemokine domain of CX3CL1 or CX3CL1.44 and full-length US28 were expressed using the BacMam system, and the ligand-receptor complexes were purified as described (*12*, *13*) with small modifications. C-terminal Fc-tagged CX3CL1 or CX3CL1.44 was secreted in the expression media, and CX3CL1-Fc or CX3CL1.44-Fc was immobilized on Protein A sepharose (GE Healthcare). Cell membrane expressed N-terminal FLAG-tagged US28 was isolated and solubilized in Hepes-buffered saline [HBS; 10 mM Hepes-Na (pH 7.2) and 150 mM NaCl] containing 1% (w/v) lauryl maltose neopentyl glycol (LMNG)/0.2% (w/v) cholesteryl hemisuccinate tris salt (CHS), instead of n-dodecyl-β-d-maltopyranoside (DDM)/CHS used previously, and US28 was captured by Protein A–bound CX3CL1-Fc or CX3CL1.44-Fc. The resin was washed with HBS containing 0.1% (w/v) LMNG/0.02% (w/v) CHS, and Fc-tag was cleaved on column by adding 3C protease. The elution containing either CX3CL1-US28 or US3CL1.44-US28 was further purified over the homemade anti-FLAG M1 sepharose with decreased detergent concentration to 0.01% (w/v) LMNG/0.002% (w/v) CHS and over a SEC column Superpose 6 10/300 GL (GE Healthcare) equilibrated with HBS containing 0.005% (w/v) LMNG/0.0005% (w/v) CHS.

To purify *apo* US28 and US27, the same buffer/detergent system was used. For *apo* US28, the cell pellet expressing US28 was lysed and spun down three times to make the membrane purer. The protein solubilized in LMNG/CHS was directly purified by anti-FLAG M1 resin and then eluted after extensive wash. The elution was sequentially purified by SEC twice to obtain the monomeric fraction. Full-length US27 (UniProt ID: P09703) was expressed with an N-terminal FLAG tag and a C-terminal His tag using the BacMam system, and the cell pellet was lysed once, solubilized in LMNG/CHS, and first purified with Ni–nitrilotriacetic acid affinity resin. The protein was eluted with 250 mM imidazole and further purified over the FLAG sepharose column and SEC equilibrated with HBS containing 0.005% (w/v) LMNG/0.0005% (w/v) CHS. For biochemical assay, proteins were concentrated, aliquoted, and flash-frozen with 10% (v/v) glycerol.

### Preparation of G protein and scFv16

G proteins and scFv16 for structural study were prepared as previously described (*15*, *31*). For GTP turnover assay, we further purified G proteins using mono Q 10/100 GL column (GE healthcare) to ensure the 1:1:1 stoichiometry of Gα:Gβ:Gγ chains.

### Preparation of vGPCR–G protein complexes

To prepare the complexes, 1.2 M equivalent of G protein was used for the coupling reaction with either CX3CL1-US28, CX3CL1.44-US28, or US27 in the presence of 1% (w/v) LMNG/0.1% (w/v) glyco-diosgenin (GDN)/0.1% (w/v) CHS. Residual GDP was hydrolyzed with apyrase. The complexes were then purified over a FLAG column and eluted in HBS containing 0.001% (w/v) LMNG/0.001% (w/v) GDN/0.0001% (w/v) CHS, 5 mM EDTA, and FLAG peptide (0.2 μg/ml). A total of 0.1 mM tris(2-carboxyethyl)phosphine (TCEP) was added immediately after protein elution. For biochemical assays, the scFv16-free vGPCR–G protein complexes were purified over a Superdex 200 10/300 GL column equilibrated with HBS containing 0.001% (w/v) LMNG, 0.001 (w/v) GDN, 0.0001% (w/v) CHS, and 0.1 mM TCEP (SEC buffer), and peak fractions were concentrated, supplemented with 10% (v/v) glycerol, and flash-frozen in aliquots.

For the 3D cryo-EM analysis, 1.2 M equivalent of scFv16 was added to the FLAG elutions containing either CX3CL1-US28-Gi, US27-Gi, or CX3CL1-US28-G11iN18 and incubated on ice for 2 hours. The mixtures were loaded onto a Superdex 200 10/300 GL equilibrated with SEC buffer, and the peak fractions were concentrated to ~30 mg/ml for CX3CL1-US28-Gi-scFv16, ~4 mg/ml for US27-Gi-scFv16, and ~25 mg/ml for CX3CL1-US28-G11iN18-scFv16. For the 2D cryo-EM analysis, scFv16-free CX3CL1-US28-Gq and CX3CL1.44-US28-Gq were purified over the SEC in the same buffer and concentrated to ~5 mg/ml.

### Tryptophan FSEC assay

Fifty microliters of 1 μM scFv16-free CX3CL1-US28-Gi, US27-Gi, or M2R-Go was loaded onto a Superdex 200 10/300 GL column attached to a JASCO FP-2020 Plus fluorescence detector equilibrated with SEC buffer, and the elution profiles were monitored by Trp-fluorescence (wavelengths for excitation at 280 nm and emission at 340 nm). To check the stabilities of the complexes in a physiological guanine-nucleotide concentration (*14*), frozen aliquots of the GPCR-Gi/o complexes were dissolved in SEC buffer and analyzed by FSEC equilibrated with SEC buffer supplemented with 36 μM GTP/300 μM GDP. To check the effect of GTP and GDP separately, each Gi/o complex was preincubated in SEC buffer supplemented with either 100 μM GTPγS, 100 μM GDP, or 100 μM GTPγS/100 μM GDP for 10 min. Each reaction was loaded on the SEC column equilibrated with SEC buffer with no nucleotide.

### BODIPY-GDP and BODIPY-GTPγS binding kinetics assay

The binding kinetics were measured as previously reported (*15*). A solution of 60 nM Gi or CX3CL1-US28-Gi was each prepared in HBS containing 0.01% (w/v) LMNG, 0.001% (w/v) CHS, 10 mM MgCl_2_, and 0.1 mM TCEP. Spectra were measured using a Fluorolog-3 spectrofluorometer (Horiba Jobin Yvon Inc.) at room temperature with wavelengths for excitation at 495 nm (0.5-nm slit size) and emission at 508 nm (10-nm slit size). Data were collected for 100 s for baseline stabilization, and either 1 μM BODIPY-GTPγS or BODIPY-GDP was added to the sample in the cuvette while the measurements were manually interrupted for 10 s. Fluorescence changes were monitored for a total of 600 s with an integration time of 1 s. The experiments were done as triplicates, and the curves were fitted using GraphPad Prism 8.

### GTP turnover assay

GTP turnover assay was performed using the GTPase-Glo Assay kit (Promega) with modified buffers and protocol from the manufacturer. The reaction was started by mixing purified receptor and G protein in an assay buffer containing 20 mM Hepes-Na (pH 7.5), 100 mM NaCl, 0.01% (w/v) LMNG, 0.001% (w/v) CHS, 10 mM MgCl_2_, 0.1 mM TCEP, 10 μM GTP, and 10 μM GDP. Activities of US28 were first tested at 0.2 μM with or without 0.25 μM CX3CL1 against either 0.5 μM G11 or Gi. Activities of US28 and US27 were tested at 0.2, 0.5, and 1 μM against 0.5 μM Gi. Activities of μOR were tested at 0.2, 0.5, 1, and 2 μM against 0.5 μM Gi. μOR was preincubated with equimolar Met-enkephalin for the receptor activation, and the reaction was done in the buffer containing 2 μM Met-enkephalin.

After incubation of receptors with G proteins at room temperature for 120 min, reconstituted GTPase-Glo reagent was added to the sample and incubated for 30 min at room temperature. Luminescence was measured following the addition of the detection reagent and incubation for 10 min at room temperature using a SpectraMax Paradigm plate reader. Data were analyzed by one-way analysis of variance with Tukey’s test using GraphPad Prism 8, and the statistical significance was indicated as *P* < 0.0001 (****). The activity of the recombinant US28 was confirmed by the ability to activate G11 and Gq with the GTP turnover assay.

### Cryo-EM grid preparation and data collection

For CX3CL1-US28-Gi-scFv16 and CX3CL1-US28-G11iN18-scFv16, 3.5 μl of the sample was applied to glow-discharged 200-mesh gold grids (Quantifoil R1.2/1.3) with the addition of 0.05% (w/v) *N*-octyl-β-d-glucopyranoside. Excess sample was blotted away by filter paper for 1 s before plunge-freezing using a Vitrobot Mark IV (Thermo Fisher Scientific) at 18°C and 100% humidity. For CX3CL1-US28-Gi-scFv16, the initial attempts for structural determination were not successful using a Titan Krios with no energy filter, limiting the overall resolution to ~4 Å but with preferred particle orientation (too thin ice). For the formal analysis, cryo-EM movies were collected using a Titan Krios operated at 300 kV equipped with K2 Summit camera and a Gatan imaging filter (GIF) at a nominal magnification of ×165,000 in superresolution counting mode, corresponding to a physical pixel size of 0.82 Å. Each movie was recorded for a total of 8 s with 0.2-s exposure per frame at an exposure rate of ~7 electrons per pixel per second and the defocus range between −1.0 and −2.0 μm, using SerialEM (*34*) with beam-image shift to collect nine images from nine holes per stage shift and focus. For CX3CL1-US28-G11iN18-scFv16, data were collected using a Titan Krios operated at 300 kV equipped with K3 camera at a nominal magnification of ×29,000 in superresolution counting mode, corresponding to a physical pixel size of 0.85 Å. Each movie was recorded for a total of 2.5 s with 0.05-s exposure per frame at an exposure rate of ~20 electrons per pixel per second and the defocus range between −1.0 and −2.0 μm using SerialEM. Cryo-EM grids for CX3CL1-US28-Gq and CX3CL1.44-US28-Gq were prepared similarly using a Vitrobot Mark IV, and data were collected as summarized in fig. S10C.

To prepare US27-Gi-scFv16 specimens, 10% volume of the SEC buffer containing either 0.1% (w/v) (1H, 1H, 2H, 2H-perfluorooctyl)-β-d-maltopyranoside (fOM), 0.1% (w/v) fOM and 1 mM GDP, or 0.5% (w/v) digitonin was added to the sample. Each mixture (3.0 μl) was applied to glow-discharged 300-mesh gold grids (Quantifoil R1.2/1.3). Excess sample was blotted to a filter paper for 3 s before plunge-freezing using a Leica EM GP (Leica Microsystems) at 16°C and 95% humidity. The cryo-EM movies were collected on the same microscope equipped with the GIF described above but with an updated K3 camera in superresolution counting mode. Magnification was set to ×105,000, corresponding to a physical pixel size of 0.8677 Å. Movies were recorded for a total of 2.5 s (fOM) or 3 s (digitonin) with 0.05-s exposure per frame at an exposure rate of ~20 electrons per pixel per second and the defocus range between −0.8 and −2.0 μm, using SerialEM with beam-image shift to collect 9 or 18 images from nine holes per stage shift and focus.

### Cryo-EM data processing

For the CX3CL1-US28-Gi-scFv16 dataset, the following scheme was applied to generate the 3D reconstructions. A total of 4546 movies were subjected to beam-induced motion correction using MotionCor2 (*35*) on the GUI of Relion version 3.1 (*36*). The contrast transfer function (CTF) parameters of the motion-corrected micrographs were estimated from the dose-weighted averages of all frames using Gctf v1.06 (*37*). A reference-based auto-picking was performed to extract 1,348,802 particles for classification. After iterative rounds of 2D classifications, selected particles were used to generate the 3D initial model and subjected to 3D classification, where two distinct 3D classes were identified and selected for refinement. The final 143,691 and 92,818 particles were used for the C-state and OC-state CX3CL1-US28-Gi-scFv16, respectively, and refined iteratively using Relion 3D auto-refine followed by per-particle CTF refinement and Bayesian polishing. We report the nominal resolutions of 3.5 and 3.6 Å for the C-state and OC-state, respectively, as determined by gold-standard Fourier shell correlation (FSC) using the 0.143 cutoff. For CX3CL1-US28-G11iN18-scFv16, the same data processing scheme was applied, from an initial 795,403 particles to a final 126,645 particles, yielding the 4.0-Å nominal resolution map. Cryo-EM 2D class averages for CX3CL1-US28-Gq and CX3CL1.44-US28-Gq and a low-resolution 3D reconstruction for CX3CL1-US28-Gq were obtained as summarized in fig. S10C.

For the US27-Gi-scFv16 datasets, data curation and processing were performed using cryoSPARC (*38*) unless otherwise noted. Low-quality movies were discarded by assessing MotionCor2-corrected micrographs generated by the SLAC movie processing pipeline, resulting in four final datasets (sample preparation batch #1: 6134 and 2503 movies for the specimens prepared with fOM and digitonin, respectively; preparation batch #2: 3596 and 6573 movies for the specimens prepared with fOM and fOM + GDP, respectively, with no apyrase treatment during the coupling reaction). For the MotionCor2-corrected micrographs from the sample preparation batch #1, preprocessing was performed. Patched CTF values were estimated for each micrograph, and initial auto-picking was done with 2D templates generated using a preliminary 3D reconstruction during sample screening (~200 micrographs from a screening microscope; FEI Tecnai F20). Particles were extracted for several rounds of 2D classifications, multiclass ab initio 3D reconstructions, and heterogeneous 3D refinements to select particles with a well-defined class. Those particles were subjected to a 3D classification without alignment using Relion version 3.1, which identified two distinct classes termed the CL-state (major population of good particles) and the OCL-state (minor population). Further heterogeneous and local nonuniform 3D refinements generated the preliminary CL-state (220,254 particles) and OCL-state (36,398 particles) maps as 3D templates. For the curated raw movies, patch motion correction was performed with binning to a physical pixel size of 0.8677 Å using default parameters, and patch CTFs were estimated. Particle picking was done using the 2D templates generated using the preliminary CL-state map, and a total of 12,254,340 “particles” including nonprotein features and buffer regions were extracted with binning to 2.0347 Å per pixel and a binned box size of 128 pixels. Several rounds of 2D classifications, multiclass ab initio 3D reconstructions, and heterogeneous 3D refinements resulted in 1,159,618 particles that appeared to be intact US27-Gi-scFv16 complexes. The specimen preparation batch #2 showed more dissociation between US27 and Gi-scFv16, which was mostly removed during the initial 2D class classification steps. The selected particles were reextracted with a physical pixel size and an unbinned box size of 300 pixels, subjected to several rounds of heterogeneous 3D refinements using the CL-state map, OCL-state map, and two junk maps generated during the preprocessing. After additional heterogeneous 3D refinement and two-class ab initio 3D reconstructions, the final subsets of 268,877 and 78,170 particles were used for iterative nonuniform 3D refinements with the fitting of per-particle CTF values. Nominal overall resolutions of the final reconstructions were 3.1 and 3.8 Å for the CL-state and the OCL-state, respectively, determined by gold-standard FSC with the 0.143 criterion. Local resolutions were estimated using Phenix (*39*). UCSF pyem v0.5 (*40*) was used to convert the cryoSPARC particles into the Relion format.

### Model building and refinement

For the CX3CL1-US28-Gi-scFv16 complexes, the crystal structure of CX3CL1-US28 (from PDB ID: 4XT1, CX3CL1-US28-Nb7) and the cryo-EM structure of Gi-scFv16 (from PDB ID: 6DDE, μOR-Gi-scFv16) were docked into the cryo-EM maps on UCSF chimera (*41*), and rigid body real-space refinements were done using Phenix. For the CL-state US27-Gi-scFv16 structure, a homology model for US27 was generated on the SWISS-MODEL server (swissmodel.expasy.org) using the US28 structure (~40% identity within TMs, from PDB ID: 4XT1) as a template. The homology model and the Gi-scFv16 (from PDB ID: 6DDE) structure were placed into the CL-state cryo-EM density map in the same way described above. For CX3CL1-US28-G11iN18-scFv16, the crystal structures of CX3CL1-US28 (from PDB ID: 4XT1), a homology model of GDP-bound G11iN18 based on the crystal structure of the Gq heterotrimer (PDB ID: 3AH8), and the cryo-EM structure of scFv16 (from PDB ID: 6DDE) were similarly docked into the density. The models were then built and refined iteratively using Coot (*42*) for manual model building and fixing, ISOLDE in UCSF ChimeraX (*43*) for model idealization, and Phenix for real-space refinement. For the OCL-state US27-Gi-scFv16, the refined CL-state US27 structure, GDP-bound inactive Gi structure (PDB ID: 1GP2), and scFv16 were docked into the map similarly as described. The structure was manually corrected using Coot with truncation of a majority of the US27 side chains and a part of extracellular regions, modeling of polyalanine intracellular loops, and then refined with Phenix using the CL-state US27 structure as a reference model except for the final refinement. For the US28 modeling, only sharpened maps were used. For the US27 modeling, unsharpened maps, the CL-state local anisotropic sharpened map, and uniform sharpened maps were used, and related maps were deposited to EMDB. All five models were lastly refined in the uniform sharpened maps. The structures and maps were visualized using UCSF Chimera, UCSF ChimeraX, and PyMol (Schrödinger LLC). All secondary structures in the figures were assigned using DSSP.

### MD simulations

We performed MD simulations using both the C- and OC-state structures of the CX3CL1-US28-Gi complexes as starting points. From each of the two structures, we initiated 12 simulations (~5 μs each) in which no artificial forces were present during the production phase and 6 simulations (~1 μs each) in which the proteins were weakly restrained to the initial structure throughout the simulations (see the “Simulation protocols” section). We also performed MD simulations using the GDP-bound CX3CL1-US28-G11iN18 complex as a starting point and, additionally, of the same complex without GDP. For each condition, we initiated 12 simulations (~5 μs each).

#### Simulation setup

For all simulations, structures were prepared using Maestro (Schrödinger LLC). ScFv16 was removed from the coordinates, and the missing parts of the N terminus of Gαi and the C terminus of Gγ were modeled in and lipidated as follows: Gαi Gly2 was myristoylated, Gαi Cys3 was palmitoylated, and Gγ Cys68 was prenylated (*44*). Simulations of Gα11iN18 were prepared analogously. Except for the C termini of Gα and Gβ, for which the complete sequences were retained in structures and simulations, neutral acetyl and methylamide groups were added to cap the N and C termini, respectively, of the protein chains. Water molecules were placed inside US28 using Dowser (*45*). Titratable residues were prepared using PROPKA (*46*) for pH 7.0. All aspartic acid and glutamic acid residues were negatively charged except for residues Asp^79^ (D^2.50^) and Asp^128^ (D^3.49^) of US28 that were manually protonated (neutral), based on evidence that they are protonated in active class A GPCRs (*47*, *48*). All arginine and lysine residues were positively charged. Histidine residues were modeled as neutral, with a hydrogen atom bound to either the delta nitrogen or the epsilon nitrogen, depending on which tautomeric state optimized the local hydrogen bond network. The systems were aligned on the receptor of the orientations of proteins in membranes (*49*) entry for US28 in complex with the chemokine domain of human CX3CL1 (PDB ID: 4XT3) (*12*). The protein complex was placed in a preequilibrated palmitoyl-oleoyl-phosphatidylcholine membrane bilayer and solvated in a water box using Dabble (*50*). Sodium and chloride ions were added such that the entire system was charge-neutral at a salt concentration of 150 mM. Water box dimensions were chosen to obtain an initial system size of 135 Å in the two membrane dimensions (width) and a height of 150 Å for the Gi complex simulations and a width of 130 Å and a height of 170 Å for the G11iN18 complex simulations.

#### Simulation protocols

In all simulations, the CHARMM36m force field was used for proteins, lipids, and ions and the TIP3P model for water molecules (*51*). Simulations were run using the AMBER18 software (*52*) under periodic boundary conditions with the Compute Unified Device Architecture version of Particle-Mesh Ewald Molecular Dynamics on one GPU (graphics processing unit) (*53*). Initial velocities of the atoms were assigned randomly for each simulation. The systems were heated from 0 to 100 K over 12.5 ps in an NVT ensemble and then from 100 to 310 K over 125 ps in an NPT ensemble at 1 bar. During the heating process, harmonic restraints with a force constant of 10.0 kcal mol^−1^ Å^−2^ were imposed on all nonhydrogen atoms of the proteins (except lipidated residues) as a harmonic potential on the deviation from their starting positions. The systems were then equilibrated at 310 K in an NPT ensemble at 1 bar, using the same form of harmonic restraints, but with the force constant starting at 5.0 kcal mol^−1^ Å^−2^ and decreasing by 1.0 kcal mol^−1^ Å^−2^ in a stepwise fashion every 2 ns for 10 ns and then by 0.1 kcal mol^−1^ Å^−2^ in a stepwise fashion every 2 ns for 18 ns. Except for the restrained simulations described below, no restraints were applied during production simulations, which were performed in the NPT ensemble at 310 K and 1 bar, using a Langevin thermostat with a collision frequency of 1 ps^−1^ for temperature coupling and a Monte Carlo barostat for semi-isotropic pressure coupling (coupling the two dimensions of the membrane plane separately from the membrane normal). For these simulations, a time step of 4 fs was used, which was enabled by the use of hydrogen mass repartitioning (*54*). The lengths of all bonds to hydrogen atoms were constrained using the SHAKE algorithm (*55*). Nonbonded interactions were cut off at 9.0 Å. Long-range electrostatic interactions were computed using the particle mesh Ewald (PME) method (*53*) with an Ewald coefficient (β) of approximately 0.31 Å and B-spline interpolation of order 4. The PME grid size was chosen such that the width of a grid cell was approximately 1 Å. Snapshots of the trajectory were saved every 200 ps.

In the absence of restraints during production simulation, the periodic boundary conditions favor a flat membrane. Restrained simulations followed the same protocol as outlined above, except that 0.01 kcal mol^−1^ Å^−2^ restraints were applied throughout the production simulation on the same protein atoms as during equilibration.

#### Simulation analysis

The AmberTools20 CPPTRAJ package (*56*) was used to reimage trajectories to a step width of 1 ns, while Visual Molecular Dynamics (*57*) was used for visualization. RMSD and root mean square fluctuation (RMSF) were calculated using MDAnalysis (*58*). RMSF was calculated with all frames aligned to the receptor backbone. RMSD curves were smoothed using a moving average with a window size of 100 ns. Plots were generated using Matplotlib.

## Supplementary Materials

### This PDF file includes:

Figs. S1 to S12 Table S1 References

## Notes

View/request a protocol for this paper from *Bio-protocol*.
