# Supplementary material for: Atypical structural snapshots of human cytomegalovirus GPCR interactions with host G proteins
Source: Sci Adv. 2022 Jan 21;8(3):eabl5442. doi: 10.1126/sciadv.abl5442 (PMC8782444; doi:10.1126/sciadv.abl5442)
Supplement: Supplementary file 1 — Figs. S1 to S12 Table S1 References [file sciadv.abl5442_sm.pdf]

Science Advances
AAAS

Supplementary Materials for

Atypical structural snapshots of human cytomegalovirus GPCR interactions with host G proteins

Naotaka Tsutsumi, Shoji Maeda*, Qianhui Qu, Martin Vögele, Kevin M. Jude, Carl-Mikael Suomivuori, Ouliana Panova, Deepa Waghray, Hideaki E. Kato, Andrew Velasco, Ron O. Dror, Georgios Skiniotis, Brian K. Kobilka, K. Christopher Garcia*

*Corresponding author. Email: shojim@umich.edu (S.M.); kcgarcia@stanford.edu (K.C.G.)

Published 21 January 2022, *Sci. Adv.* **8**, eabl5442 (2022)
DOI: 10.1126/sciadv.abl5442

This PDF file includes:

Figs. S1 to S12
Table S1
References

Supplementary Figures

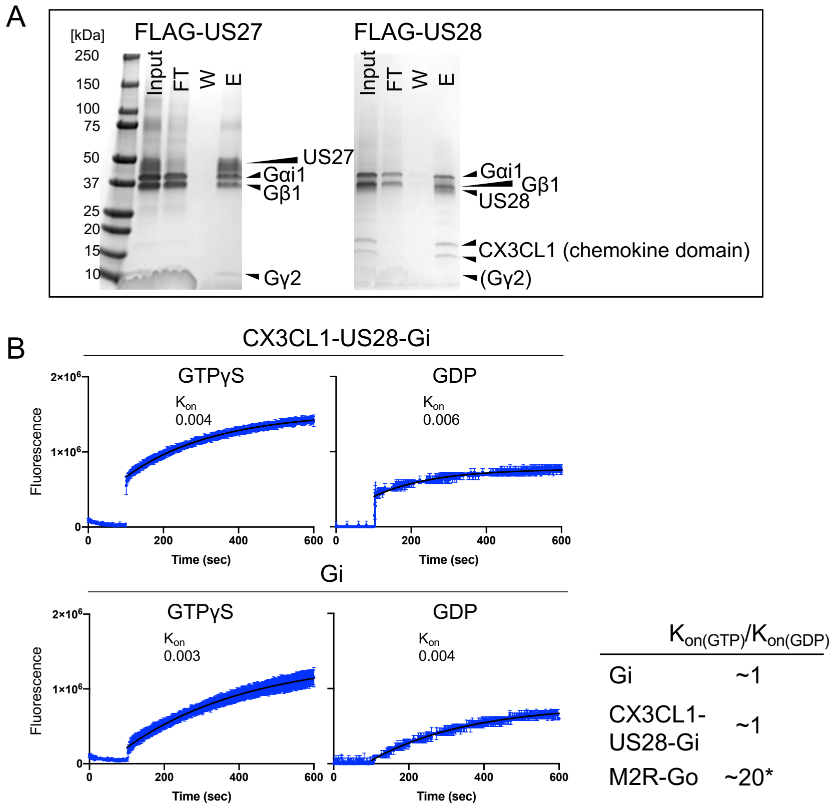

Fig. S1 Gi heterotrimer pulled-down with the vGPCRs and nucleotide binding kinetics to Gi in the presence or absence of CX3CL1-US28.

(A) FLAG affinity pull-down for FLAG-tagged US27 or US28 after Gi coupling reactions for large scale complex preparations. US28 was prepared with its ligand CX3CL1 for protein stability. For each the vGPCR, Input, FT: flowthrough, W: wash, and E: elution were run on SDS-PAGEs. (B) BODIPY-guanine nucleotide binding assay for free Gi heterotrimer and CX3CL1-US28-Gi. Binding kinetics were measured for either GDP or GTPγS, and compared with the published M2R-Go data* (*15*). Data were shown as the fitting curves to the mean values (black lines) with SD (blue error bars). The GTP/GDP binding preferences are shown by the ratio of the association constants to GTP and GDP, K_{on}(GTP)/K_{on}(GDP).

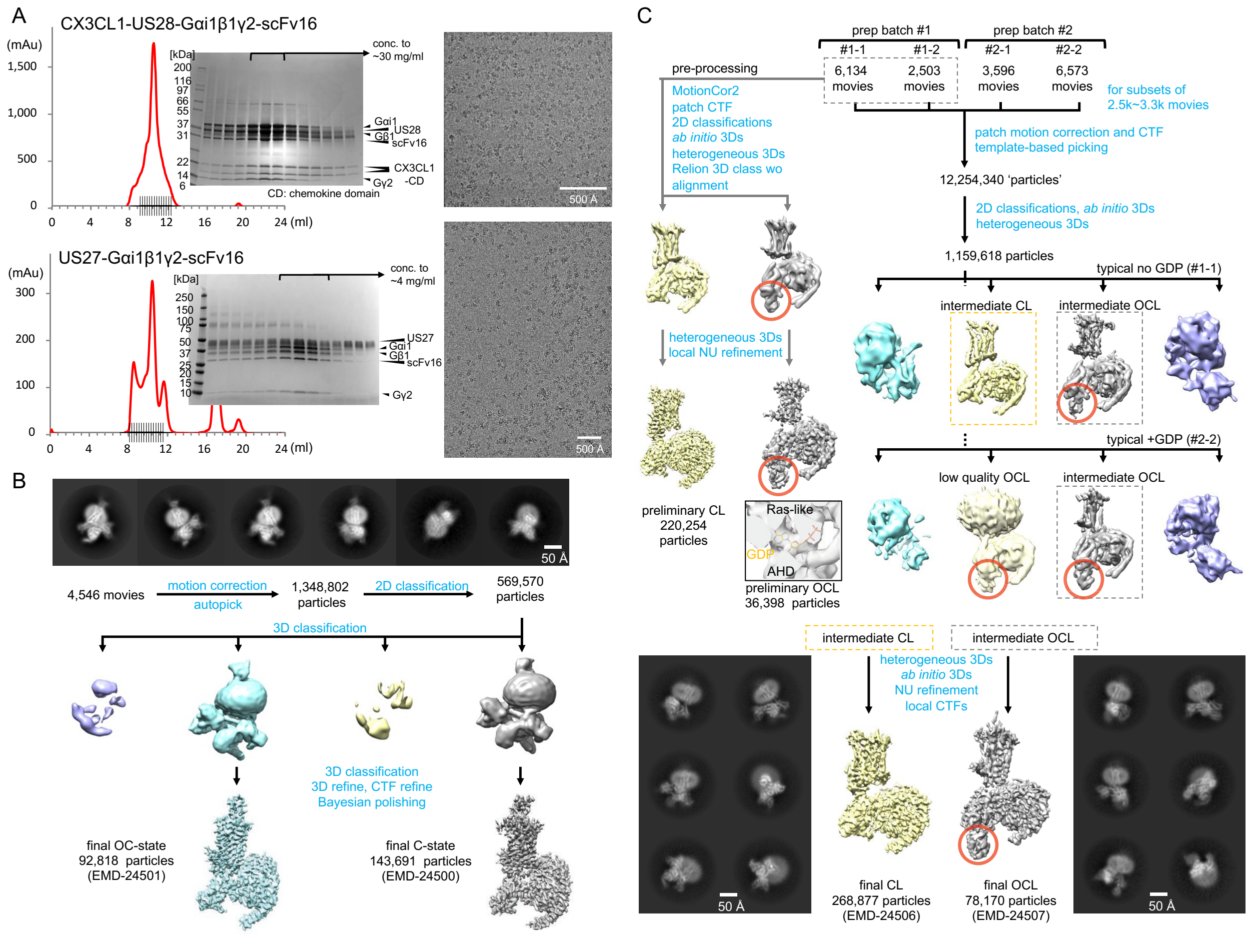

Fig. S2 Cryo-EM sample preparation, data collection, processing, and analysis.
(A) The SEC profiles, SDS-PAGEs, and representative cryo-EM micrographs for CX3CL1-US28-Gi-scFv16 and US27-Gi-scFv16. The peak SEC fractions were concentrated for cryo-EM specimen preparation and indicated on the SDS-PAGEs. Data processing scheme and representative 2D class averages from different directions for (B) CX3CL1-US28-Gi-scFv16 and (C) US27-Gi-scFv16. For US27-Gi-scFv16 maps, α-helical domain (AHD) densities are marked by red circles, and a GDP density first observed in the preliminary OCL-state map is highlighted in a box with a stick model of a GDP molecule.

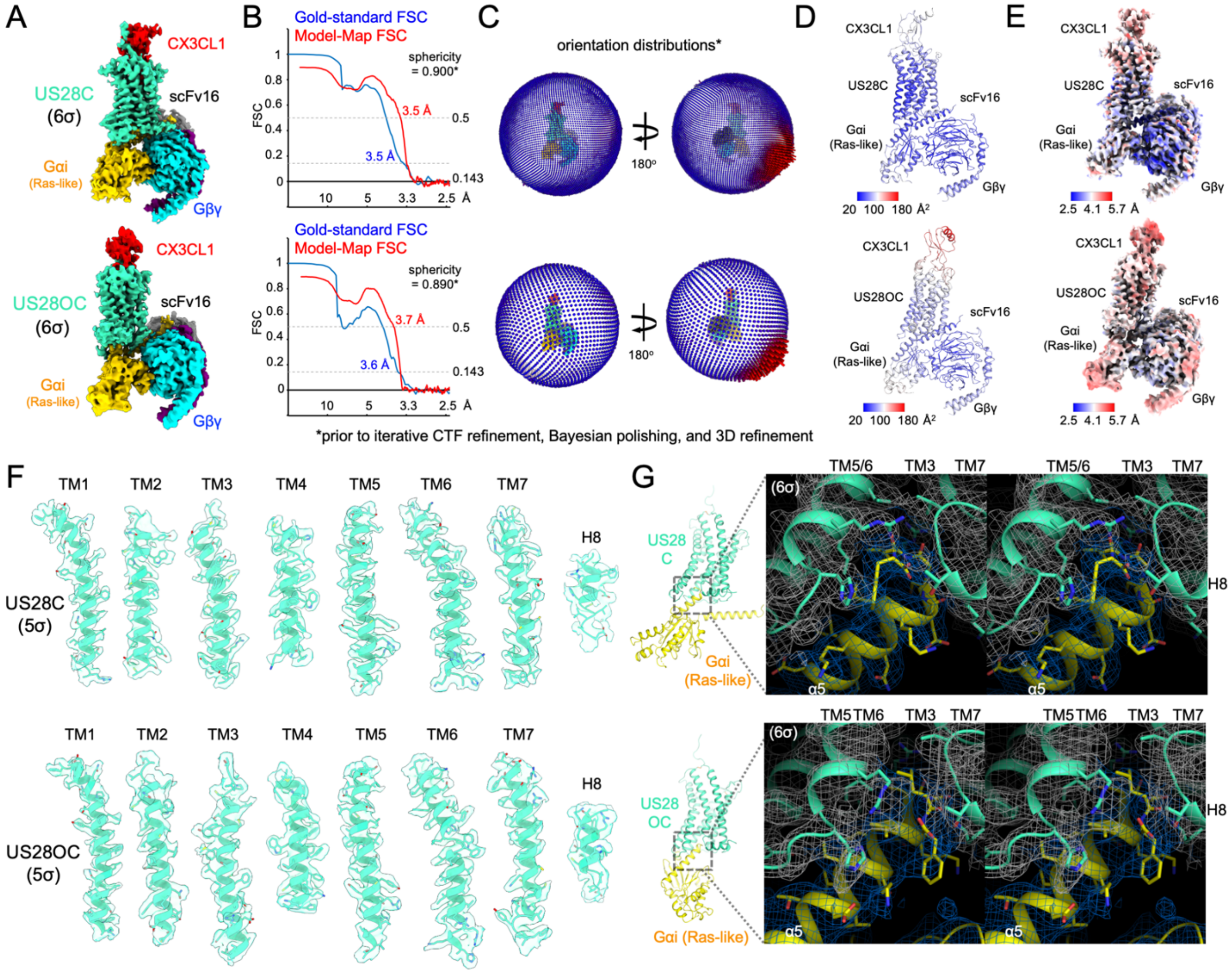

Fig. S3 Analysis of cryo-EM 3D models for CX3CL1-US28-Gi-scFv16 in the C- and OC-states.
(A) The overall 3D maps for CX3CL1-US28-Gi-scFv16 in the C-state (US28C, top) and the OC-state (US28OC, bottom), colored by chains in green-cyan (US28), gold (Gα), cyan (Gβ), purple (Gγ), and gray (scFv16). The map contour levels are set to 6σ. (B) Gold-standard FSC curves (blue line, corrected with masks) and model-map FSC curves (red line, auto-masked) for the 3D maps and the final models of (top) US28C and (bottom) US28OC. The gold-standard FSC curves are the output of the final Relion postprocessing (*36*), and the map sphericities were calculated on the 3DFSC server (*59*) (3dfsc.salk.edu). Other part of the panels were prepared using Phenix (*39*). (C) 3D histograms overlaid on the 3D maps show the Euler angle distributions used in the 3D refinements for (top) US28C and (bottom) US28OC. The height and color from blue to red indicate the relative number of particles from the specific direction. The asterisks (*) in the panels B and C indicate the numbers or plots generated based on the 3D refinements before iterative CTF refinements, Bayesian polishing, and 3D refinements due to the lack of final half maps and 3D alignment files. For this reason, we report (D) B-factor plots on the cartoon models, and (E) full map-based local resolution estimation by DeepRes (*60*) on 3D maps. (F) The local 3D maps overlaid on the individual TMs and H8 for US28C and US28OC. The map contour levels are set to 5σ. (G) Stereoviews of the cryo-EM densities depicted around Gαi-α5 bound to US28. The map contour levels are set to 6σ.

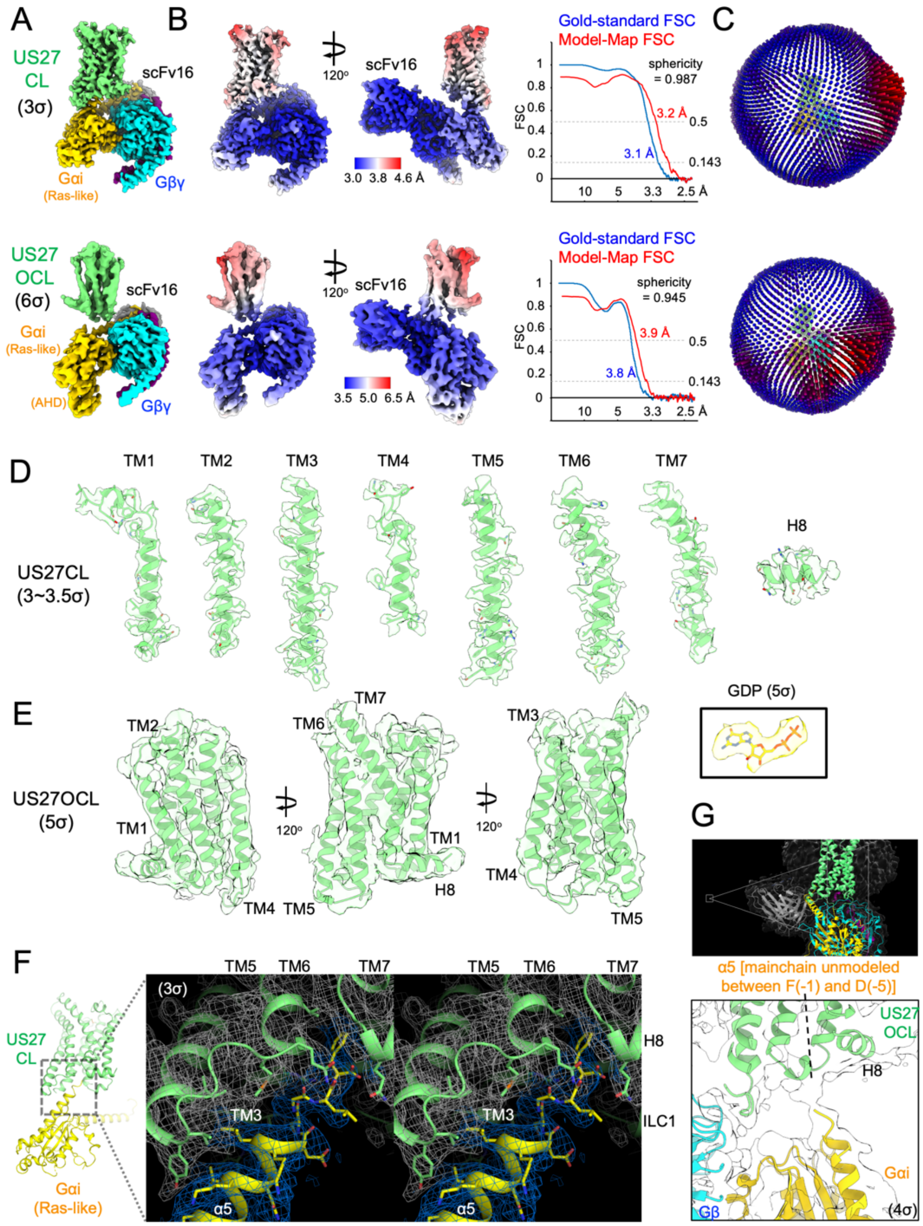

Fig. S4 Analysis of cryo-EM 3D maps and models for US27-Gi-scFv16 in the CL- and OCL-states.

(A) The 3D reconstructions of US27-Gi-scFv16 in the CL-state (US27CL, top) and the OCL-state (US27OCL, bottom), colored by chains in lime (US27), gold (Gα), cyan (Gβ), purple (Gγ), and gray (scFv16). (B) Local resolution estimates (blue-white-red colored on the maps), gold-standard FSC curves (blue line, corrected with auto-masks), and model-map FSC curves (red line, auto-masked) for the final 3D maps and models of (top) US27CL and (bottom) US27OCL generated using Phenix. The non-uniform local resolutions, with the higher resolution observed in the G protein part, indicate the flexibility between US27 and Gi. The map sphericities were calculated using the 3DFSC server. (C) 3D histograms overlaid on the 3D maps show the Euler angle distributions used in the final refinements of (top) US27CL and (bottom) US27OCL. The height and color from blue to red indicate the relative number of particles from the specific direction. The map contour levels are set to 3σ for US27CL and 5σ for US27OCL. (D) The local 3D maps overlaid on the individual TMs and H8 for US27CL (3-3.5σ), or the 7TM domain for US27OCL (5σ). The GDP observed between the Ras-like domain and AHD of Gαi is shown in close up in the outlined box with the cryo-EM density at 5σ contour level. (F) A stereoview of the cryo-EM density for the CL-state US27 complex, showing the interface between Gαi-α5 and US27. The cryo-EM map contour level is set to 3σ. (G) The blurred cryo-EM density of the Gαi-α5 approaching intracellular pocket of US27-7TM. The cryo-EM contour level is set low to 4σ.

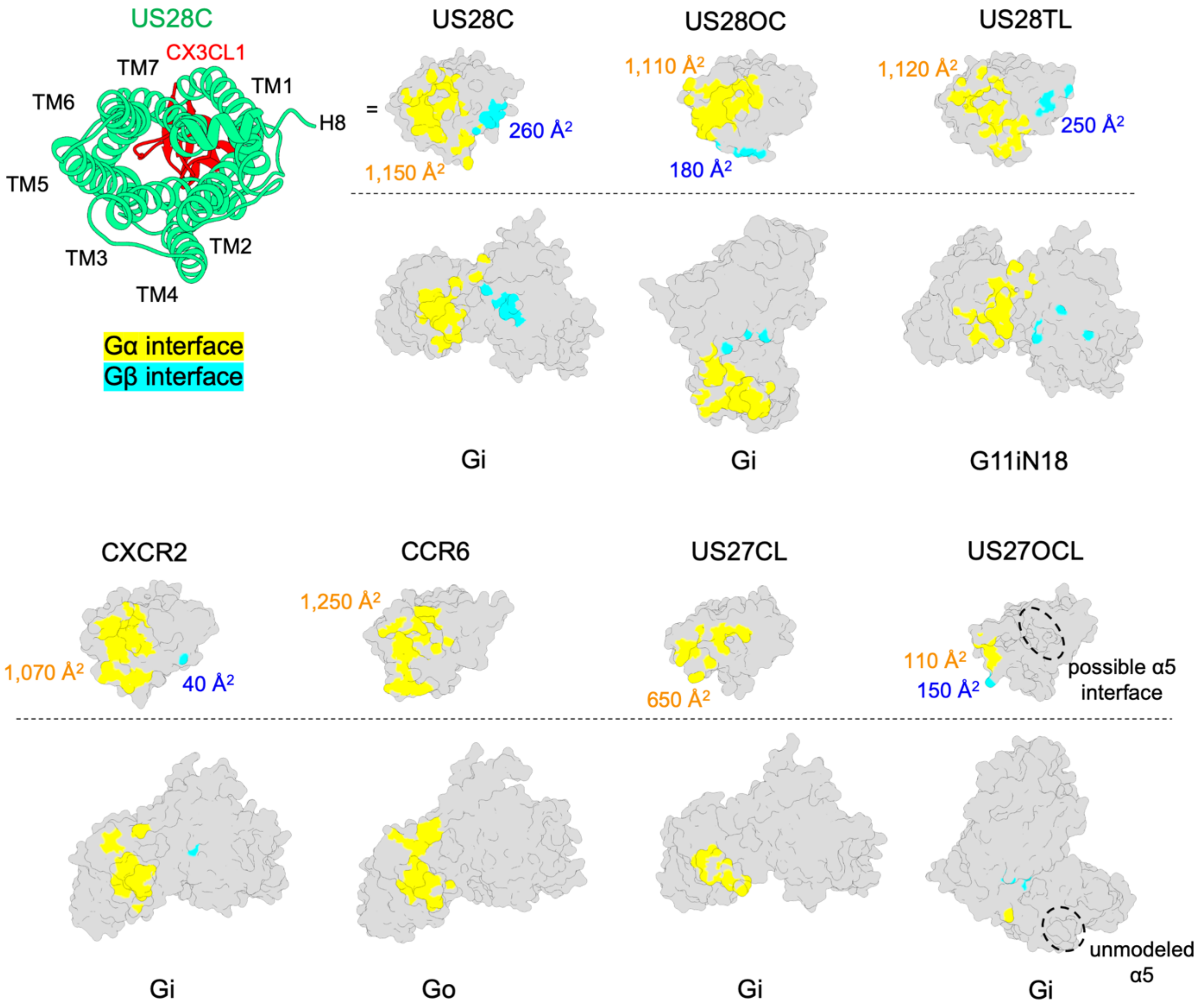

Fig. S5 Footprint analysis of the vGPCR-G protein interface.

Opened interfaces of the vGPCR-G protein complexes analyzed in this study. CXCR2-Gi (PDB ID: 6LFO) and CCR6-Go (PDB ID: 6WWZ) interfaces are included for comparison. The receptor and G protein heterotrimers are shown as surface representation, colored with the footprint of the interface surfaces. GPCR:Gα interface is colored in yellow, and GPCR:Gβ interface is colored in cyan. All the receptor structures are aligned to the cartoon model of the CX3CL1-US28 complex in the C-state shown at the top-left. The US27-Gi interface area is underestimated due to conservative modeling for US27 sidechains overall and at the interface, especially with the unmodeled Gαi-α5 (fig. S4G) marked with dashed circles. The figure was prepared using UCSF Chimera.

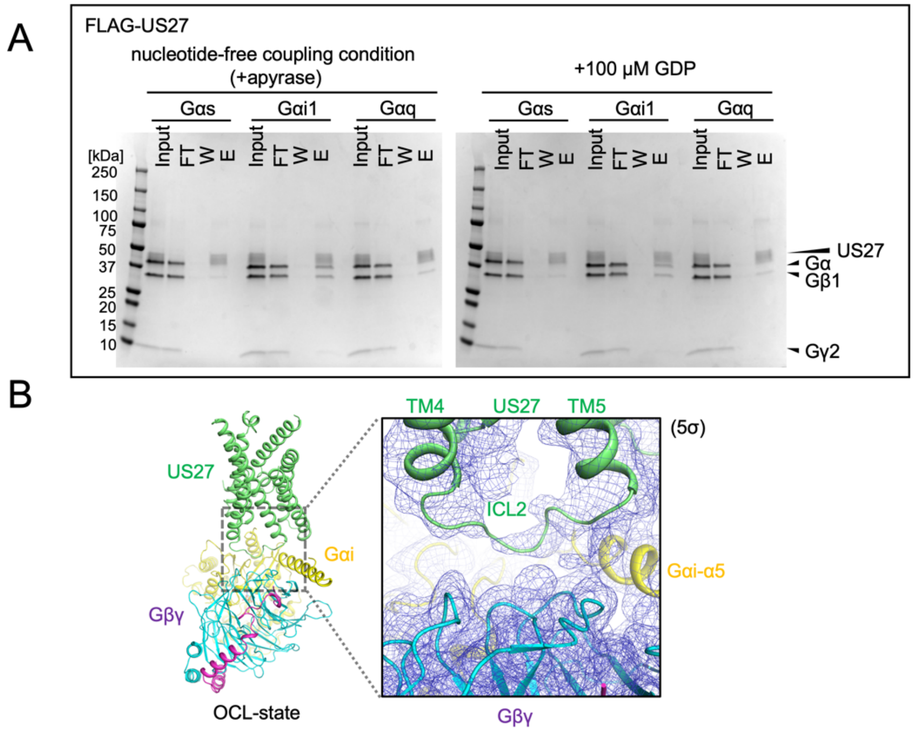

Fig. S6 G protein binding selectivity of US27.
(A) FLAG-US27 pull-down for either Gs, Gi or Gq in the absence or presence of GDP. For each condition, Input, FT: flowthrough, W: wash, and E: elution were run on SDS-PAGEs. US27 preferentially binds Gi, but weaker interactions were still observed between US27 and Gs or Gq probably because of (B) additional contacts between US27-ICL2 and Gβ subunit. The map contour level is set to 5σ.

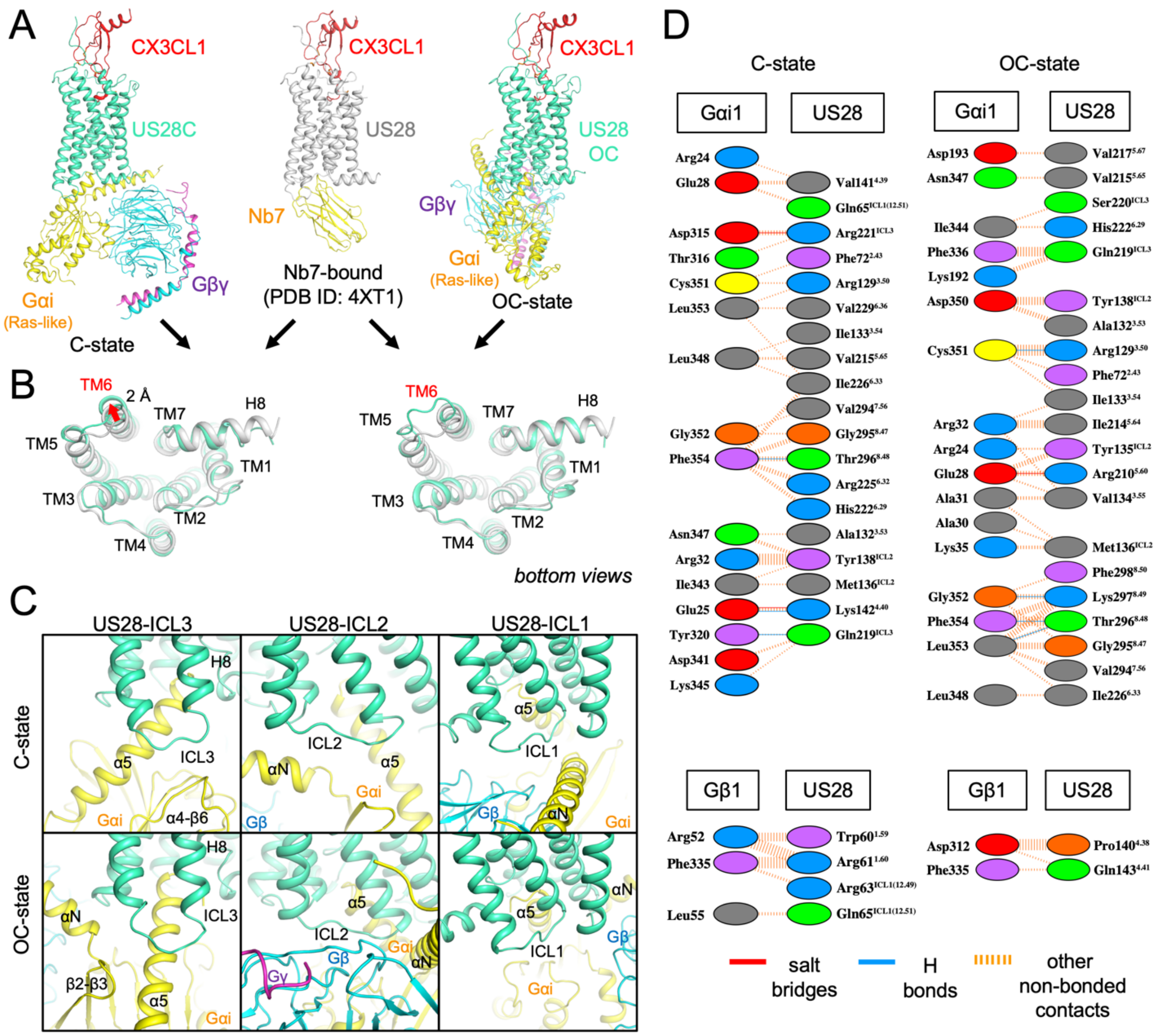

Fig. S7 Comparison of the US28 structures.
(A) Comparison of the US28 structures bound to Gi or the G protein mimetic nanobody Nb7 (PDB ID: 4XT1), with the whole complexes displayed side-by-side. (B) Superimposition of US28 bound to Nb7 with (left) US28C and (right) US28OC. (C) Close-up views of the interfaces between US28-ICLs and Gi in their C- and OC-states. Each cartoon model is colored in red (CX3CL1), green-cyan (US28C and US28OC), gray (US28 bound to Nb7), yellow (Gαi1 and Nb7), cyan (Gβ1), and purple (Gγ2). (D) The residue-residue interaction network for US28 in (left) the C-state and (right) the OC-state, displayed using PDBsum (ebi.ac.uk/pdbsum). The cartoons are colored in blue (positively charged amino acids; His, Lys, and Arg), red (negatively charged; Asp, Glu), green (neutral polar; Ser, Thr, Asn, Gln), yellow (Cys), gray (aromatic; Phe, Tyr, Trp), purple (aliphatic; Ala, Val, Leu, Ile, Met), and orange (Pro and Gly).

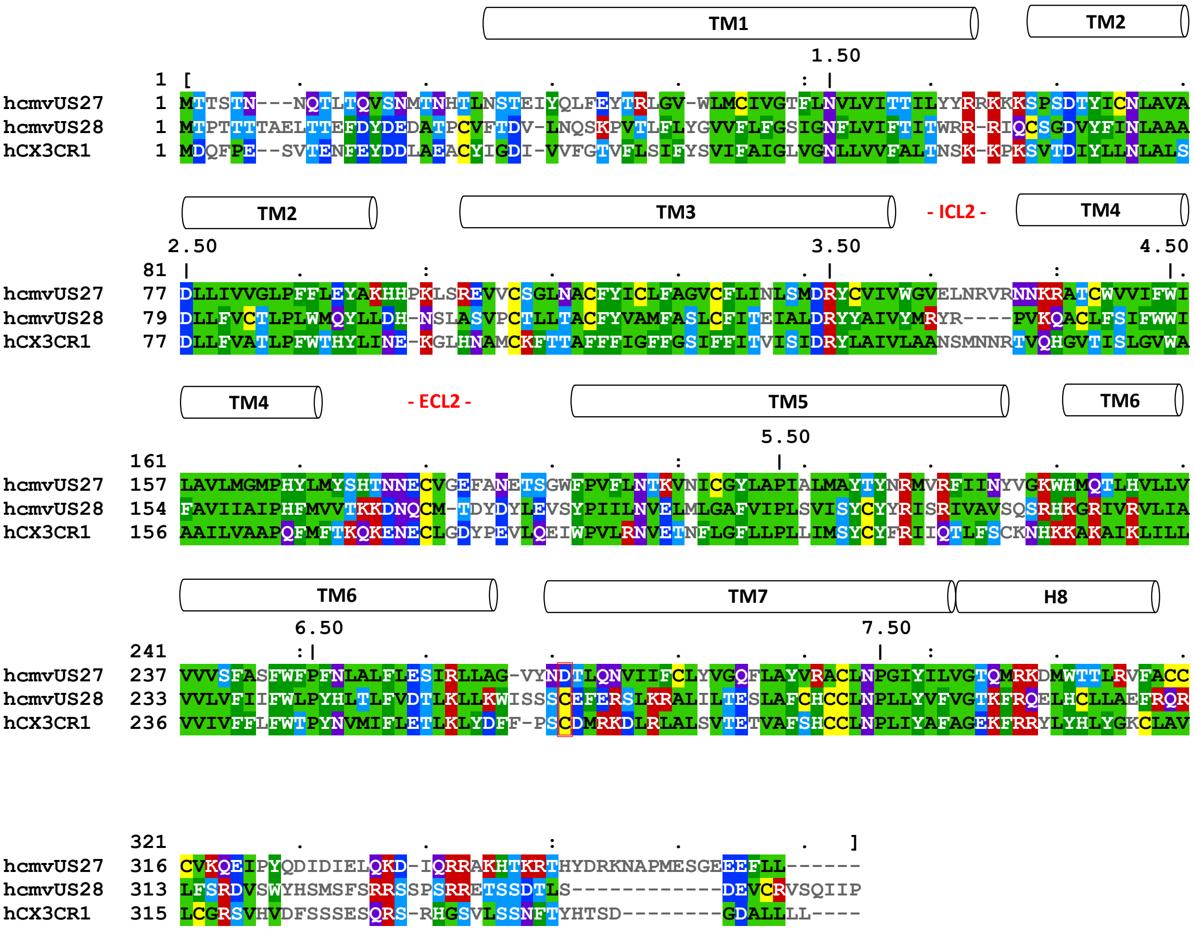

Fig. S8 Multiple sequence alignment of HCMV US27 and US28, and human CX3CR1.
The structure-based sequence alignment was performed on the PROMALS3D server using the three GPCR sequences and the two vGPCR structures in complex with Gi as inputs, and the result was visualized using MView. TM cartoons for US28 are displayed above the alignment as a marker.

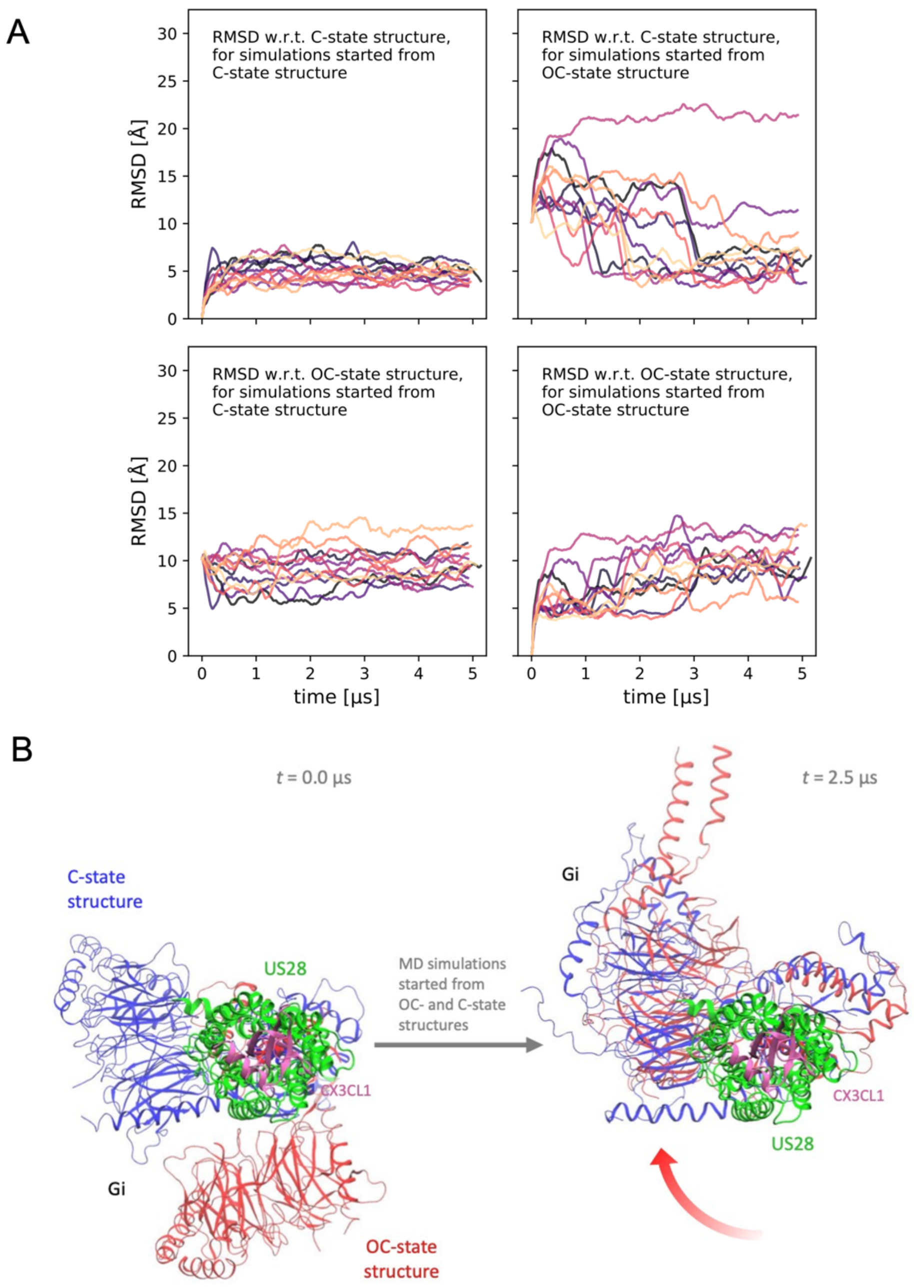

Fig. S9 Analysis of the MD simulations performed for the scFv16-free CX3CL1-US28-Gi complex.
(A) RMSD of Gαi-α5 in simulations of the CX3CL1-US28-Gi complex in a flat lipid membrane. For each unbiased (unrestrained) simulation, we show the root mean squared deviation (RMSD) of Gαi-α5 (Cα atoms of residues 329 to 354) with respect to (w.r.t.) the C-state (top) and OC-state (bottom) cryo-EM structures. Simulation frames are aligned on US28 (Cα atoms of transmembrane helices 1 to 4). Simulations started from the C-state cryo-EM structure are shown at left, and those started from the OC-state structure are shown at right. In 10 of 12 simulations started from the OC-state structure, Gαi-α5 transitioned fully or partially to the C-state within 5 µsec, whereas in all 12 simulations started from the C-state structure, Gαi-α5 remained in the C-state. (B) Lateral re-orientation of Gi in MD simulations of CX3CL1-US28-Gi in a flat lipid membrane. In molecular dynamics simulations started from the OC-state cryo-EM structure, Gi (red) rotates by approximately 90°, adopting an orientation more similar to that of the C-state structure. In simulations started from the C-state structure, Gi maintains its orientation relative to the receptor. All simulation frames were aligned on US28. US28 and CX3CL1 are shown with fixed coordinates for simplicity.

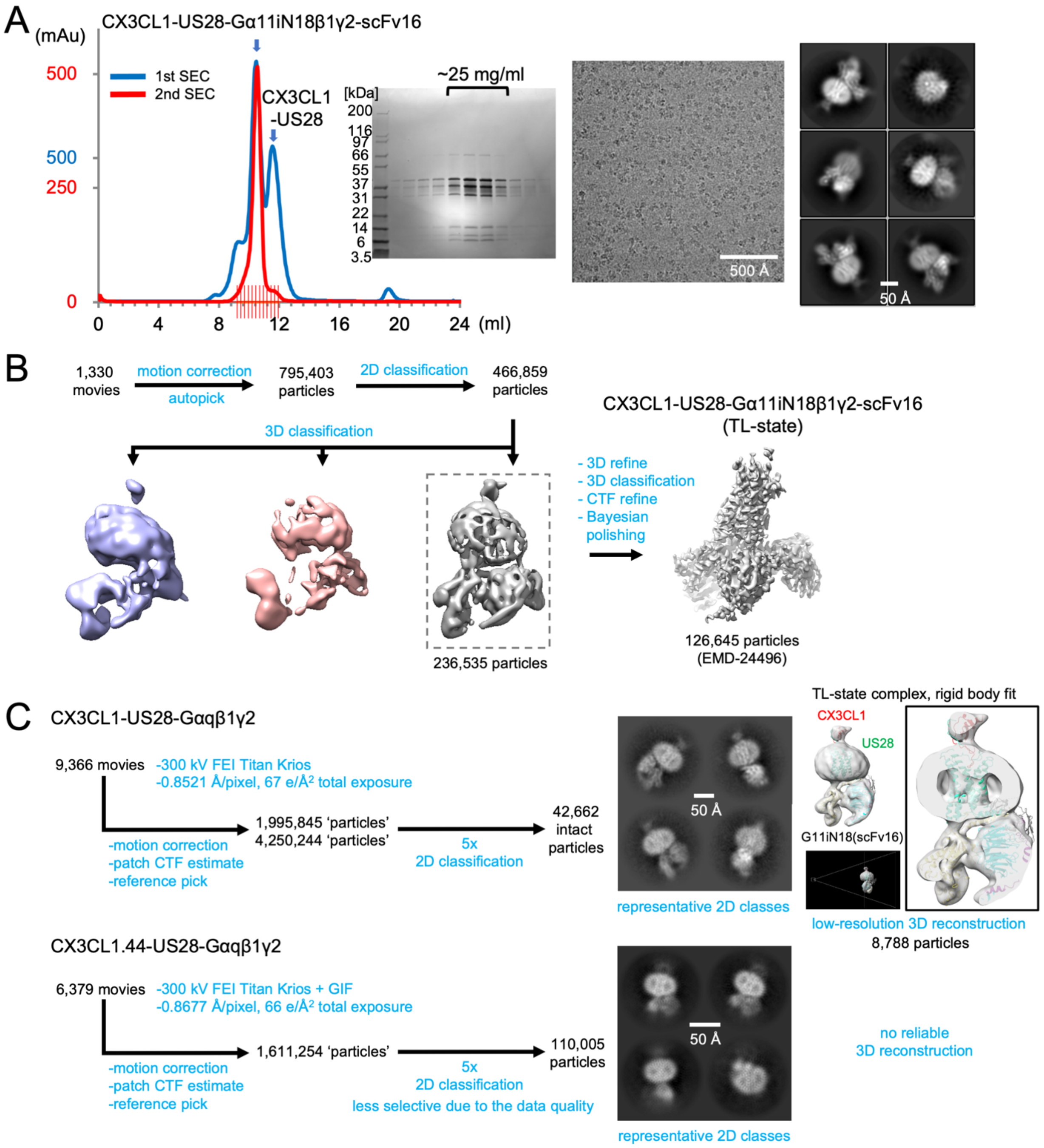

Fig. S10 Purification and cryo-EM analysis for US28-Gq/11 complexes.
(A) SEC profiles, SDS-PAGEs, representative cryo-EM micrographs, and 2D classes for CX3CL1-US28-G11iN18-scFv16. The peak fractions of the second SEC run were used for cryo-EM analysis. (B) Cryo-EM data processing scheme and 3D classification for CX3CL1-US28-G11iN18-scFv16. (C) Cryo-EM experiments and representative 2D class averages for CX3CL1-US28-Gq and CX3CL1.44-US28-Gq without scFv16. For the complex with the wild-type ligand, we were able to obtain a low-resolution 3D reconstruction enabling rigid body docking of the TL-state CX3CL1-US28-G11iN18-scFv16 structure into the cryo-EM map. In addition to the clear AHD density observed, the receptor-G protein docking geometry of the CX3CL1-US28-G11 complex is consistent with the TL-state structure with the chimeric G11 and scFv16.

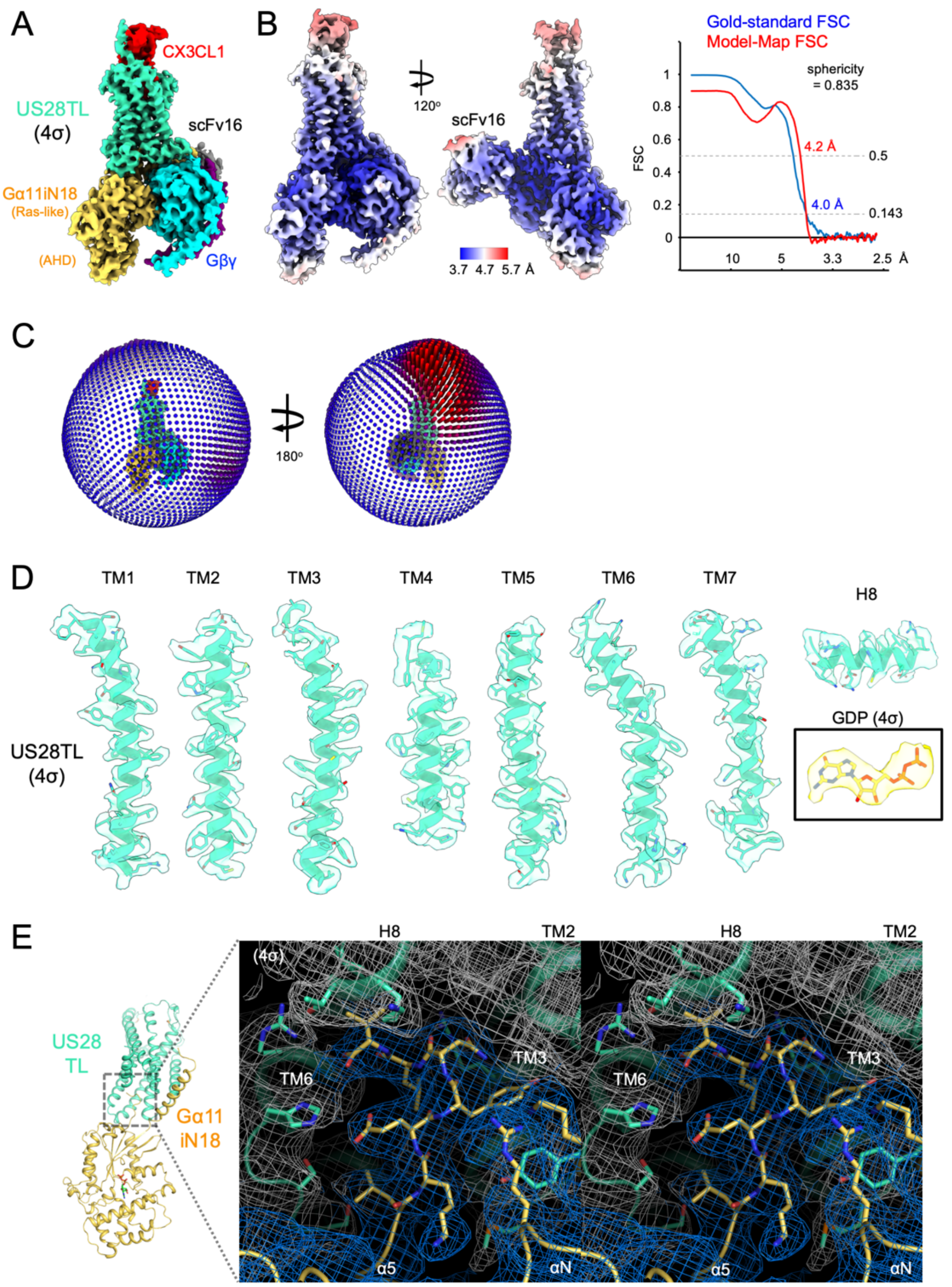

Fig. S11 Analysis of cryo-EM 3D models for the TL-state CX3CL1-US28-G11iN18-scFv16.
(A) The overall 3D map of CX3CL1-US28-G11iN18-scFv16 in the TL-state (US28TL), colored by chains in green-cyan (US28), yellow-orange (Gα), cyan (Gβ), purple (Gγ), and gray (scFv16). The map contour level is set to 4σ. (B) Local resolution estimate (blue-white-red colored on the map), gold-standard FSC curve (blue line, corrected with an auto-mask), and model-map FSC curve (red line, auto-masked) for the final 3D map and model generated using Phenix. The map sphericity was calculated using the 3DFSC server. (C) The Euler angle distribution of the final 3D reconstruction shown by 3D histograms overlaid on the 3D map. The height and color from blue to red indicate the relative number of particles from the specific direction. (D) The local 3D maps overlaid on the individual TMs and H8 for US28TL with the map contour levels set to 4σ. The GDP observed between the Ras-like domain and AHD of Gαi is shown in close up in the outlined box with the cryo-EM density at 4σ contour level. (E) A stereoview of the cryo-EM densities at the primary US28-Gα11iN18 interface. The map contour levels are set to 4σ.

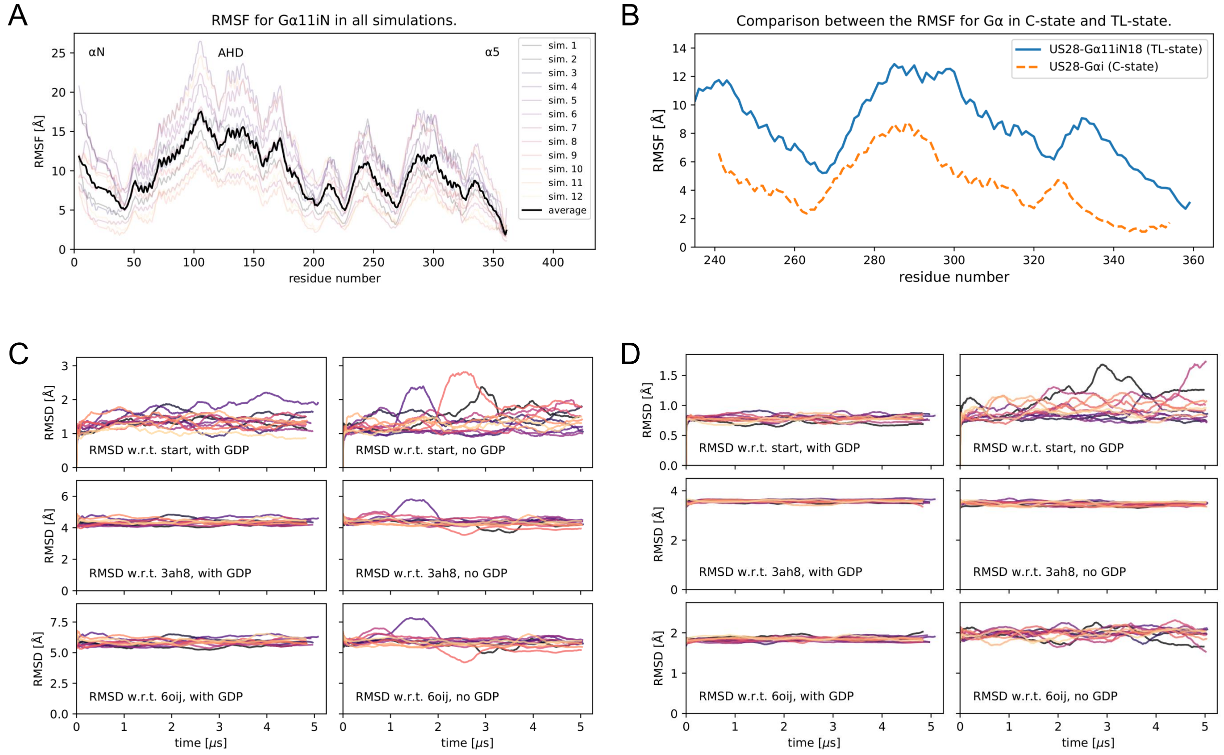

Fig. S12 Analysis of the MD simulations performed for the TL-state CX3CL1-US28-G11iN18 complex without scFv16. (A, B) Flexibility of CX3CL1-US28-G11iN18 in MD simulations. (A) RMSF plot of Gα11iN18 for the simulations of the TL-state CX3CL1-US28-G11iN18 bound to GDP, showing the relative stability of the C-terminal anchor region of Gα11iN18. The bold black line indicates the average RMSF used to color Gα11iN18 of Fig. 7H. (B) Comparison of average RMSF plots of Gα11iN18 for the simulation of the TL-state CX3CL1-US28-G11iN18 and of Gαi for the simulation of the C-state CX3CL1-US28-Gi, showing the higher mobility of the Gα chain in the TL-state. All simulations were performed without the scFv16. **(C, D) Structural comparison of the MD simulations with or without GDP.** (C) RMSD plots of the α5 region when aligned to the β-barrel in the Ras-like domain of Gα, with respect to the inactive (in complex with YM-25489034, PDB ID: 3AH8) and active (in complex with muscarinic acetylcholine receptor M1 (M1R), PDB ID: 6OIJ) conformations. (D) RMSD plots of the guanine nucleotide-binding site with respect to (w.r.t.) the starting model (start), as well as the inactive (PDB ID: 3AH8) and active (PDB ID: 6OIJ) conformations.

Table S1 Cryo-EM data collection and refinement statistics

	CX3CL1-US28-Gαi1β1γ2-scFv16		US27-Gαi1β1γ2-scFv16		CX3CL1-US28-Gα11iN18β1γ2-scFv16
	C-state	OC-state	CL-state	OCL-state	TL-state
**Data collection and processing**					
Nominal magnification	165,000x		105,000x		29,000x
Calibrated magnification	60,976x		57,624x		58,679x
Voltage (kV)	300		300		300
Electron exposure (e/Å^2)	83		66 (+fOM) or 80 (+digitonin)		67
Defocus range (μm)	-1.0 to -2.0		-0.8 to -2.0		-1.0 to -2.0
Pixel size (Å)	0.82		0.8677		0.85
Symmetry imposed	C1		C1		C1
Initial "particle" images (no.)	1,348,802		12,254,340		795,403
Final particle images (no.)	143,691	92,818	268,877	78,170	126,645
Map resolution (Å)	3.5	3.6	3.1	3.8	4.0
FSC threshold	0.143	0.143	0.143	0.143	0.143
Map resolution range (Å)	2.5 to 5.7*	2.5 to 5.7*	3.0 to 4.6	3.5 to 6.5	3.7 to 5.7
**Refinement**					
EMDB code	EMD-24500	EMD-24501	EMD-24506	EMD-24507	EMD-24496
PDB code	7RKM	7RKN	7RKX	7RKY	7RKF
Initial model used (PDB ID)	4XT1, 6DDE	4XT1, 6DDE	4XT1, 6DDE	CL-state complex, 1GP2	4XT1, 3AH8, 6DDE
Model resolution (Å)	3.5	3.7	3.3	3.9	4.2
FSC threshold	0.5	0.5	0.5	0.5	0.5
Map sharpening *B* factor (Å^2)	-150	-150	-20 or LocalAniso**	-20	-150
Model composition					
Non-hydrogen atoms	9,491	9,393	8,559	8,894	10,563
Protein residues	1,201	1,201	1,126	1,219	1,334
Ligands	1 (CLR)	N/A	N/A	1 (GDP)	2 (GDP, NAG)
B factors (Å^2)					
Protein	45.08	69.83	69.02	74.63	66.38
Ligand	40.87 (CLR)	N/A	N/A	55.98 (GDP)	147.09 (NAG), 45.85 (GDP)
R.m.s. deviations					
Bond lengths (Å)	0.005	0.004	0.005	0.004	0.005
Bond angles (°)	0.960	0.970	0.710	0.800	0.758
Validation					
MolProbity score	1.66	1.75	1.45	1.58	1.77
Clashscore	7.47	7.74	4.01	5.43	7.64
Poor rotamers (%)	0	0	0	0	0
Ramachandran plot					
Favored (%)	96.28	95.35	96.11	95.83	94.84
Allowed (%)	3.72	4.65	3.89	4.17	5.16
Disallowed (%)	0	0	0	0	0
EMRinger score	3.32	2.81	4.19	2.95	2.38

*Full map-based estimation by DeepRes. **Local anisotropic sharpening by Phenix.

REFERENCES AND NOTES

1. J. W. Griffith, C. L. Sokol, A. D. Luster, Chemokines and chemokine receptors: Positioning cells for host defense and immunity. *Annu. Rev. Immunol.* **32**, 659–702 (2014).

2. S. Montaner, I. Kufareva, R. Abagyan, J. S. Gutkind, Molecular mechanisms deployed by virally encoded G protein–coupled receptors in human diseases. *Annu. Rev. Pharmacol. Toxicol.* **53**, 331–354 (2013).

3. M. J. Cannon, D. S. Schmid, T. B. Hyde, Review of cytomegalovirus seroprevalence and demographic characteristics associated with infection. *Rev. Med. Virol.* **20**, 202–213 (2010).

4. J. A. Scarborough, J. R. Paul, J. V. Spencer, Evolution of the ability to modulate host chemokine networks via gene duplication in human cytomegalovirus (HCMV). *Infect. Genet. Evol.* **51**, 46–53 (2017).

5. T. Frank, I. Niemann, A. Reichel, T. Stamminger, Emerging roles of cytomegalovirus-encoded G protein-coupled receptors during lytic and latent infection. *Med. Microbiol. Immunol.* **208**, 447–456 (2019).

6. T. Imai, K. Hieshima, C. Haskell, M. Baba, M. Nagira, M. Nishimura, M. Kakizaki, S. Takagi, H. Nomiyama, T. J. Schall, O. Yoshie, Identification and molecular characterization of fractalkine receptor CX3CR1, which mediates both leukocyte migration and adhesion. *Cell* **91**, 521–530 (1997).

7. B. A. Krishna, W. E. Miller, C. M. O'Connor, US28: HCMV's swiss army knife. *Viruses* **10**, 445 (2018).

8. P. Casarosa, R. A. Bakker, D. Verzijl, M. Navis, H. Timmerman, R. Leurs, M. J. Smiti, Constitutive signaling of the human cytomegalovirus-encoded chemokine receptor US28. *J. Biol. Chem.* **276**, 1133–1137 (2001).

9. J. Randolph-Habecker, B. Rahill, B. Torok-Storb, J. Vieira, P. E. Kolattukudy, B. H. Rovin, D. D. Sedmak, The expression of the cytomegalovirus chemokine receptor homolog US28 sequesters biologically active cc chemokines and alters IL-8 production. *Cytokine* **19**, 37–46 (2002).

10. B. Cambien, M. Pomeranz, H. Schmid-Antomarchi, M. A. Millet, V. Breittmayer, B. Rossi, A. Schmid-Alliana, Signal transduction pathways involved in soluble fractalkine-induced monocytic cell adhesion. *Blood* **97**, 2031–2037 (2001).

11. T. Flock, A. S. Hauser, N. Lund, D. E. Gloriam, S. Balaji, M. M. Babu, Selectivity determinants of GPCR-G-protein binding. *Nature* **545**, 317–322 (2017).

12. J. S. Burg, J. R. Ingram, A. J. Venkatakrishnan, K. M. Jude, A. Dukkipati, E. N. Feinberg, A. Angelini, D. Waghray, R. O. Dror, H. L. Ploegh, K. C. Garcia, Structural basis for chemokine recognition and activation of a viral G protein–coupled receptor. *Science* **347**, 1113–1117 (2015).

13. T. F. Miles, K. Spiess, K. M. Jude, N. Tsutsumi, J. S. Burg, J. R. Ingram, D. Waghray, G. M. Hjorto, O. Larsen, H. L. Ploegh, M. M. Rosenkilde, K. C. Garcia, Viral GPCR US28 can signal in response to chemokine agonists of nearly unlimited structural degeneracy. *eLife* **7**, e35850 (2018).

14. T. W. Traut, Physiological concentrations of purines and pyrimidines. *Mol. Cell. Biochem.* **140**, 1–22 (1994).

15. S. Maeda, A. Koehl, H. Matile, H. Hu, D. Hilger, G. F. X. Schertler, A. Manglik, G. Skiniotis, R. J. P. Dawson, B. K. Kobilka, Development of an antibody fragment that stabilizes GPCR/G-protein complexes. *Nat. Commun.* **9**, 3712 (2018).

16. H. E. Kato, Y. Zhang, H. Hu, C.-M. Suomivuori, F. M. N. Kadji, J. Aoki, K. Krishna Kumar, R. Fonseca, D. Hilger, W. Huang, N. R. Latorraca, A. Inoue, R. O. Dror, B. K. Kobilka, G. Skiniotis, Conformational transitions of a neurotensin receptor 1–Gi1 complex. *Nature* **572**, 80–85 (2019).

17. Q. Zhou, D.-H. Yang, M. Wu, Y. Guo, W. Guo, L. Zhong, X. Cai, A. Dai, W. Jang, E. I. Shakhnovich, Z.-J. Liu, R. C. Stevens, N. A. Lambert, M. M. Babu, M.-W. Wang, S. Zhao, Common activation mechanism of class A GPCRs. *eLife* **8**, e50279 (2019).

18. V. Isberg, C. De Graaf, A. Bortolato, V. Cherezov, V. Katritch, F. H. Marshall, S. Mordalski, J. P. Pin, R. C. Stevens, G. Vriend, D. E. Gloriam, Generic GPCR residue numbers - Aligning topology maps while minding the gaps. *Trends Pharmacol. Sci.* **36**, 22–31 (2015).

19. K. Liu, L. Wu, S. Yuan, M. Wu, Y. Xu, Q. Sun, S. Li, S. Zhao, T. Hua, Z. J. Liu, Structural basis of CXC chemokine receptor 2 activation and signalling. *Nature* **585**, 135–140 (2020).

20. D. J. Wasilko, Z. L. Johnson, M. Ammirati, Y. Che, M. C. Griffor, S. Han, H. Wu, Structural basis for chemokine receptor CCR6 activation by the endogenous protein ligand CCL20. *Nat. Commun.* **11**, 3031 (2020).

21. J. Huotari, A. Helenius, Endosome maturation. *EMBO J.* **30**, 3481–3500 (2011).

22. W. Huang, M. Masureel, Q. Qu, J. Janetzko, A. Inoue, H. E. Kato, M. J. Robertson, K. C. Nguyen, J. S. Glenn, G. Skiniotis, B. K. Kobilka, Structure of the neurotensin receptor 1 in complex with β-arrestin 1. *Nature* **579**, 303–308 (2020).

23. A. Fraile-Ramos, T. N. Kledal, A. Pelchen-Matthews, K. Bowers, T. W. Schwartz, M. Marsh, The human cytomegalovirus US28 protein is located in endocytic vesicles and undergoes constitutive endocytosis and recycling. *Mol. Biol. Cell* **12**, 1737–1749 (2001).

24. A. Fraile-Ramos, A. Pelchen-Matthews, T. N. Kledal, H. Browne, T. W. Schwartz, M. Marsh, Localization of HCMV UL33 and US27 in endocytic compartments and viral membranes. *Traffic* **3**, 218–232 (2002).

25. W. E. Miller, D. A. Houtz, C. D. Nelson, P. E. Kolattukudy, R. J. Lefkowitz, G-protein-coupled receptor (GPCR) kinase phosphorylation and β-arrestin recruitment regulate the constitutive signaling activity of the human cytomegalovirus US28 GPCR. *J. Biol. Chem.* **278**, 21663–21671 (2003).

26. A. Fraile-Ramos, T. A. Kohout, M. Waldhoer, M. Marsh, Endocytosis of the viral chemokine receptor US28 does not require beta-arrestins but is dependent on the clathrin-mediated pathway. *Traffic* **4**, 243–253 (2003).

27. J. Droese, T. Mokros, R. Hermosilla, R. Schülein, M. Lipp, U. E. Höpken, A. Rehm, HCMV-encoded chemokine receptor US28 employs multiple routes for internalization. *Biochem. Biophys. Res. Commun.* **322**, 42–49 (2004).

28. K. Kim, T. Che, O. Panova, J. F. DiBerto, J. Lyu, B. E. Krumm, D. Wacker, M. J. Robertson, A. B. Seven, D. E. Nichols, B. K. Shoichet, G. Skiniotis, B. L. Roth, Structure of a hallucinogen-activated Gq-coupled 5-HT2A serotonin receptor. *Cell* **182**, 1574–1588.e19 (2020).

29. Y. Du, N. M. Duc, S. G. Rasmussen, D. Hilger, X. Kubiak, L. Wang, J. Bohon, H. R. Kim, M. Wegrecki, A. Asuru, K. M. Jeong, J. Lee, M. R. Chance, D. T. Lodowski, B. K. Kobilka, K. Y. Chung, Assembly of a GPCR-G protein complex. *Cell* **177**, 1232–1242.e11 (2019).

30. T. Frank, A. Reichel, O. Larsen, A. C. Stilp, M. M. Rosenkilde, T. Stamminger, T. Ozawa, N. Tschammer, Attenuation of chemokine receptor function and surface expression as an immunomodulatory strategy employed by human cytomegalovirus is linked to vGPCR US28. *Cell Commun. Signal* **14**, 31 (2016).

31. N. Tsutsumi, Q. Qu, M. Mavri, M. S. Baggesen, S. Maeda, D. Waghray, C. Berg, B. K. Kobilka, M. M. Rosenkilde, G. Skiniotis, K. C. Garcia, Structural basis for the constitutive activity and immunomodulatory properties of the Epstein-Barr virus-encoded G protein-coupled receptor BILF1. *Immunity* **54**, 1405–1416.e7 (2021).

32. P. Griffiths, I. Baraniak, M. Reeves, The pathogenesis of human cytomegalovirus. *J. Pathol.* **235**, 288–297 (2015).

33. T. W. M. De Groof, E. G. Elder, M. Siderius, R. Heukers, J. H. Sinclair, M. J. Smit, Viral G protein–coupled receptors: Attractive targets for herpesvirus-associated diseases. *Pharmacol. Rev.* **73**, 828–846 (2021).

34. D. N. Mastronarde, Automated electron microscope tomography using robust prediction of specimen movements. *J. Struct. Biol.* **152**, 36–51 (2005).

35. S. Q. Zheng, E. Palovcak, J. P. Armache, K. A. Verba, Y. Cheng, D. A. Agard, MotionCor2: Anisotropic correction of beam-induced motion for improved cryo-electron microscopy. *Nat. Methods* **14**, 331–332 (2017).

36. J. Zivanov, T. Nakane, B. O. Forsberg, D. Kimanius, W. J. H. Hagen, E. Lindahl, S. H. W. Scheres, New tools for automated high-resolution cryo-EM structure determination in RELION-3. *eLife* **7**, e42166 (2018).

37. K. Zhang, Gctf: Real-time CTF determination and correction. *J. Struct. Biol.* **193**, 1–12 (2016).

38. A. Punjani, J. L. Rubinstein, D. J. Fleet, M. A. Brubaker, CryoSPARC: Algorithms for rapid unsupervised cryo-EM structure determination. *Nat. Methods* **14**, 290–296 (2017).

39. D. Liebschner, P. V. Afonine, M. L. Baker, G. Bunkoczi, V. B. Chen, T. I. Croll, B. Hintze, L. W. Hung, S. Jain, A. J. McCoy, N. W. Moriarty, R. D. Oeffner, B. K. Poon, M. G. Prisant, R. J. Read, J. S. Richardson, D. C. Richardson, M. D. Sammito, O. V. Sobolev, D. H. Stockwell, T. C. Terwilliger, A. G. Urzhumtsev, L. L. Videau, C. J. Williams, P. D. Adams, Macromolecular structure

determination using x-rays, neutrons and electrons: Recent developments in Phenix. *Acta Crystallogr. D Struct. Biol.* **75**, 861–877 (2019).

40. D. Asarnow, E. Palovcak, Y. Cheng, UCSF pyem v0.5. *Zenodo* (2019); https://doi.org/10.5281/zenodo.3576630.

41. E. F. Pettersen, T. D. Goddard, C. C. Huang, G. S. Couch, D. M. Greenblatt, E. C. Meng, T. E. Ferrin, UCSF Chimera—A visualization system for exploratory research and analysis. *J. Comput. Chem.* **25**, 1605–1612 (2004).

42. P. Emsley, K. Cowtan, Coot: Model-building tools for molecular graphics. *Acta Crystallogr. D Biol. Crystallogr.* **60**, 2126–2132 (2004).

43. T. D. Goddard, C. C. Huang, E. C. Meng, E. F. Pettersen, G. S. Couch, J. H. Morris, T. E. Ferrin, UCSF ChimeraX: Meeting modern challenges in visualization and analysis. *Protein Sci.* **27**, 14–25 (2018).

44. P. B. Wedegaertner, P. T. Wilson, H. R. Bourne, Lipid modifications of trimeric G proteins. *J. Biol. Chem.* **270**, 503–506 (1995).

45. L. Zhang, J. Hermans, Hydrophilicity of cavities in proteins. *Proteins* **24**, 433–438 (1996).

46. M. H. M. Olsson, C. R. Søndergaard, M. Rostkowski, J. H. Jensen, PROPKA3: Consistent treatment of internal and surface residues in empirical pKa predictions. *J. Chem. Theory Comput.* **7**, 525–537 (2011).

47. P. Ghanouni, H. Schambye, R. Seifert, T. W. Lee, S. G. F. Rasmussen, U. Gether, B. K. Kobilka, The effect of pH on β2 adrenoceptor function. Evidence for protonation-dependent activation. *J. Biol. Chem.* **275**, 3121–3127 (2000).

48. A. Ranganathan, R. O. Dror, J. Carlsson, Insights into the role of $Asp79^{2.50}$ in β_2 adrenergic receptor activation from molecular dynamics simulations. *Biochemistry* **53**, 7283–7296 (2014).

49. M. A. Lomize, A. L. Lomize, I. D. Pogozheva, H. I. Mosberg, OPM: Orientations of proteins in membranes database. *Bioinformatics* **22**, 623–625 (2006).

50. R. Betz, Dabble (Version v2.6.3). *Zenodo* (2017).

51. J. Huang, S. Rauscher, G. Nawrocki, T. Ran, M. Feig, B. L. De Groot, H. Grubmüller, A. D. MacKerell Jr., CHARMM36m: An improved force field for folded and intrinsically disordered proteins. *Nat. Methods* **14**, 71–73 (2016).

52. T. S. Lee, D. S. Cerutti, D. Mermelstein, C. Lin, S. Legrand, T. J. Giese, A. Roitberg, D. A. Case, R. C. Walker, D. M. York, GPU-accelerated molecular dynamics and free energy methods in Amber18: Performance enhancements and new features. *J. Chem. Inf. Model.* **58**, 2043–2050 (2018).

53. R. Salomon-Ferrer, A. W. Götz, D. Poole, S. Le Grand, R. C. Walker, Routine microsecond molecular dynamics simulations with AMBER on GPUs. 2. Explicit solvent particle mesh Ewald. *J. Chem. Theory Comput.* **9**, 3878–3888 (2013).

54. C. W. Hopkins, S. Le Grand, R. C. Walker, A. E. Roitberg, Long-time-step molecular dynamics through hydrogen mass repartitioning. *J. Chem. Theory Comput.* **11**, 1864–1874 (2015).

55. J. P. Ryckaert, G. Ciccotti, H. J. C. Berendsen, Numerical integration of the cartesian equations of motion of a system with constraints: Molecular dynamics of n-alkanes. *J. Comput. Phys.* **23**, 327–341 (1977).

56. D. R. Roe, T. E. Cheatham III, PTRAJ and CPPTRAJ: Software for processing and analysis of molecular dynamics trajectory data. *J. Chem. Theory Comput.* **9**, 3084–3095 (2013).

57. W. Humphrey, A. Dalke, K. Schulten, VMD: Visual molecular dynamics. *J. Mol. Graph.* **14**, 33–38 (1996).

58. R. J. Gowers, M. Linke, J. Barnoud, T. J. E. Reddy, M. N. Melo, S. L. Seyler, J. Domański, D. L. Dotson, S. Buchoux, I. M. Kenney, O. Beckstein, MDAnalysis: A Python package for the rapid analysis of molecular dynamics simulations, in *Proceedings of the 15th Python in Science Conference*, S. Benthall, S. Rostrup, Eds. Austin, TX, 2016, pp. 98–105.

59. Y. Zi Tan, P. R. Baldwin, J. H. Davis, J. R. Williamson, C. S. Potter, B. Carragher, D. Lyumkis, Addressing preferred specimen orientation in single-particle cryo-EM through tilting. *Nat. Methods* **14**, 793–796 (2017).

60. E. Ramírez-Aportela, J. Mota, P. Conesa, J. M. Carazo, C. O. S. Sorzano, DeepRes: A new deep-learning- and aspect-based local resolution method for electron-microscopy maps. *IUCrJ*. **6**, 1054–1063 (2019).
